# Mesenchymal stem cell therapy in decompensated liver cirrhosis: a long-term follow-up analysis of the randomized controlled clinical trial

**DOI:** 10.1007/s12072-021-10199-2

**Published:** 2021-11-29

**Authors:** Ming Shi, Yuan-Yuan Li, Ruo-Nan Xu, Fan-Ping Meng, Shuang-Jie Yu, Jun-Liang Fu, Jin-Hua Hu, Jing-Xin Li, Li-Feng Wang, Lei Jin, Fu-Sheng Wang

**Affiliations:** 1 Medical Department of Infectious Diseases, Fifth Medical Center of Chinese PLA General Hospital, National Clinical Research Center for Infectious Diseases, Beijing, 100039 China; 2 Medical Department of Liver Diseases, Fifth Medical Center of Chinese PLA General Hospital, National Clinical Research Center for Infectious Diseases, Beijing, 100039 China; 3 Jiangsu Provincial Center for Disease Control and Prevention, Nanjing, 210009 Jiangsu China

**Keywords:** Mesenchymal stem cells, Umbilical cord, Decompensated liver cirrhosis, HBV infection, Long-term survival, Liver function, Adverse events, Clinical trial, Prospective open-labeled study, Randomized controlled study

## Abstract

**Background:**

Mesenchymal stem cell (MSC) infusion was reported to improve liver function in patients with decompensated liver cirrhosis (DLC); however, whether the medication can improve outcome of these patients is poorly understood.

**Methods:**

This prospective, open-labeled, randomized controlled study enrolled 219 patients with HBV-related DLC who were divided into control group (*n* = 111) and umbilical cord-derived MSC (UC-MSC)-treated group (*n* = 108), then all of them received a follow-up check from October 2010 to October 2017. The treated patients received three times of UC-MSC infusions at 4-week intervals plus conventional treatment that was only used for control group. The overall survival rate and HCC-free survival rate were calculated as primary endpoints and the liver function and adverse events associated with the medication were also evaluated.

**Results:**

During the follow-up check period from 13 to 75th months, there was a significantly higher overall survival rate in the treated group than the control group, while the difference of the hepatocellular carcinoma event-free survival rate between the treated and control groups was not observed during the 75-month follow-up. UC-MSC treatment markedly improved liver function, as indicated by the levels of serum albumin, prothrombin activity, cholinesterase, and total bilirubin during 48 weeks of follow-up. No significant side effects or treatment-related complications were observed in the UC-MSC group.

**Conclusions:**

Therapy of UC-MSC is not only well tolerated, but also significantly improves long-term survival rate, as well as the liver function in patients with HBV-related DLC. UC-MSC medication, therefore, might present a novel therapeutic approach for the disease.

**Graphic abstract:**

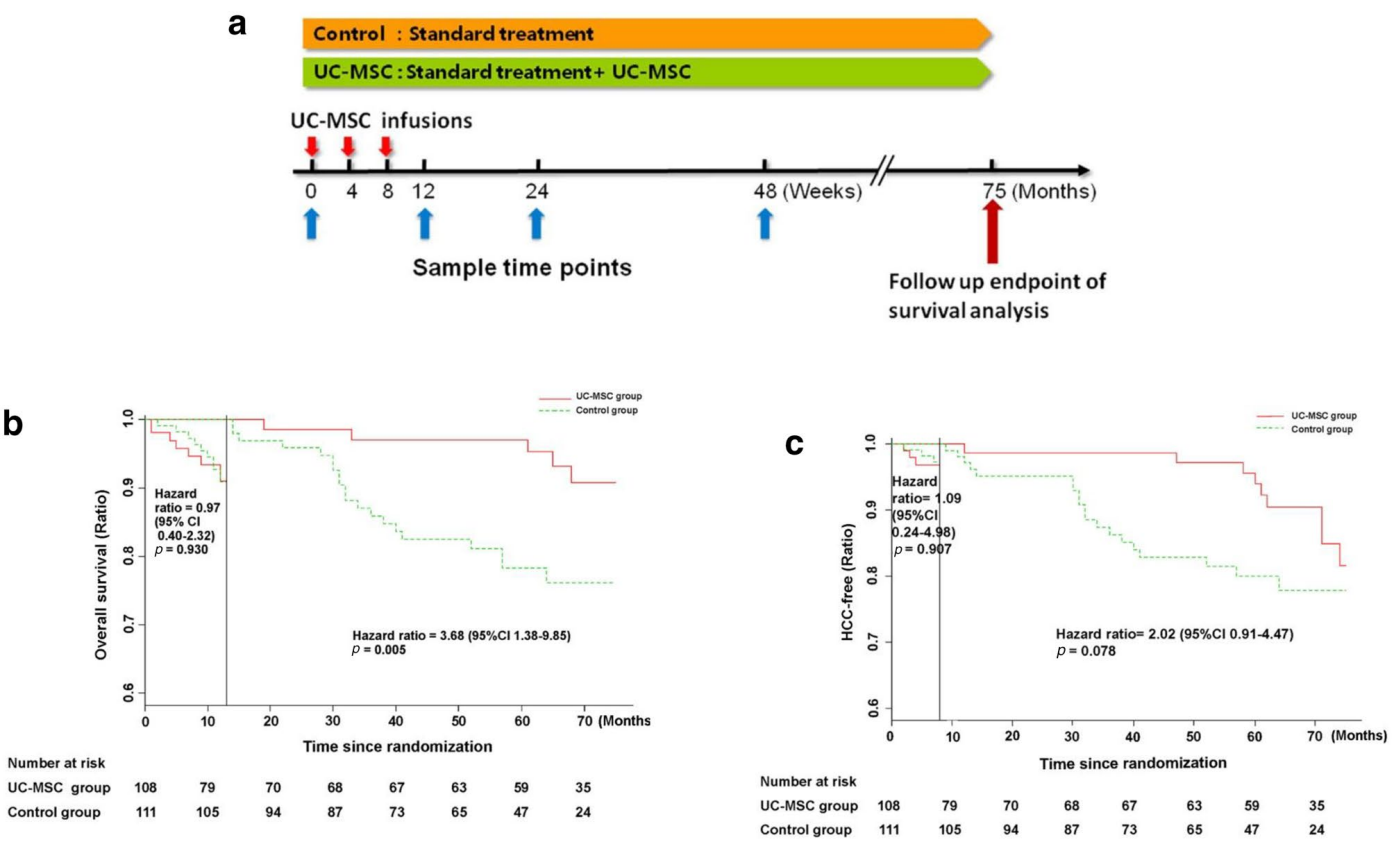

**Supplementary Information:**

The online version contains supplementary material available at 10.1007/s12072-021-10199-2.

## Introduction

In the past decade, mesenchymal stem cell (MSC) therapies have emerged as a novel alternative for the treatment of end-stage liver diseases. Studies from animal models have shown that the infusion of bone marrow-derived MSC (BM-MSC) can ameliorate liver fibrosis [1, 2] and reverse fulminant hepatic failure [3–5]. In the clinical setting, several studies have shown that infusions of autologous BM-MSC can significantly improve liver function in patients with liver cirrhosis [6, 7]. In particular, BM-MSC infusions have been demonstrated to be safe and feasible for the treatment of liver failure [8, 9]. A recent research also found that autologous BM-MSC therapy was safe in terms of improving histological fibrosis and liver function in patients with alcoholic cirrhosis [10]. Further, allogeneic MSC therapies with, for example, allogeneic BM-MSC, umbilical cord MSC (UC-MSC), and umbilical cord blood MSC, have been shown to be safe and beneficial in patients with liver cirrhosis caused by autoimmune diseases [11]. Our previous studies showed that UC-MSC infusion significantly improved liver function in patients with decompensated liver cirrhosis [12] and primary biliary cirrhosis [13] and increased the survival rates of patients with acute-on-chronic liver failure (ACLF) [14]. However, the relatively small size of the patient cohorts and the absence of evaluations of long-term efficacy (based on prognosis and safety) prevent definite conclusions from being made about the safety and efficacy of this treatment in liver diseases.

To date, very few studies have demonstrated the effectiveness of MSC treatment in patients with liver cirrhosis, and the findings are mostly based on 12-month follow-up data. A recent study with up to 10 years of follow-up demonstrated the efficacy of autologous peripheral blood stem cells in the treatment of liver cirrhosis [15]. Our previous clinical trials have shown that UC-MSC treatment improves liver function in decompensated liver cirrhosis patients and ACLF patients during 72-week follow-up; however, the long-term effect of UC-MSC treatment in these patients remains uncertain. To address these issues, in our study, the long-term efficacy and safety of UC-MSC infusions in the treatment of patients with decompensated liver cirrhosis were observed for 75 months, which is the longest follow-up period reported so far for UC-MSC treatment of this condition. The findings are expected to lay the foundation for UC-MSC as a novel therapeutic strategy for the treatment of patients with HBV-related decompensated liver cirrhosis and possibly other end-stage liver diseases.

## Patients and methods

### Study design and participants

This is a prospective, open-labeled and randomized controlled study registered at ClinicalTrial.gov (Number NCT01220492) and authorized by the General Logistic Ministry of Healthy, China. We carried out this study at a single-center, Beijing 302 hospital, in the North of China.

Decompensated liver cirrhosis is characterized by a series of clinical manifestations, including gastrointestinal bleeding, hepatic encephalopathy, jaundice and ascites, based on previously stable cirrhosis. Therefore, the criteria for enrollment of patients in this study included: manifestation of decompensated liver cirrhosis; 18–65 years of age, and positive testing for serum hepatitis B surface antigen (HBsAg) for more than 6 months, negative pregnancy test (female patients in fertile age), willing to participate in this clinical trial, able to understand and sign informed consent. The exclusion criteria were as follows: the presence of underlying neoplasms; evidence of significant extrahepatic biliary diseases; active thrombosis of the portal or hepatic veins; renal or respiratory failure; chronic HCV infection; other liver diseases including alcoholic liver disease, non-alcoholic fatty liver disease, drug-induced hepatitis, autoimmune hepatitis, Wilson disease, and hemochromatosis; diabetes mellitus; severe cardiovascular disease and drug-uncontrolled hypertension; the presence of severe comorbidities; pregnant woman; lack of a supportive family; and refusal to sign the informed consent form. All patients signed a written informed consent form in accordance with the Institutional Review Board guidelines.

### Randomization

Patients were randomly allocated to the conventional treatment (control group) or UC-MSC infusion group (UC-MSC treatment group) at a ratio of 1:1 according to a computer-generated randomization list. Before treatment, all patients were assigned a unique randomization code. The randomization system allocated patients to receive either the conventional treatment or three UC-MSC infusions at 4-week intervals plus conventional treatment. This was an open-label study. Thus, both clinicians and participants were aware of the treatment allocation.

### Procedures

UC-MSC was prepared in an approved good manufacturing practice (GMP)-compliant facility and identified as described previously [14]. In brief, with the written consent of the maternity patients, fresh human umbilical cords were obtained after birth and collected in cold Xeno-free MesenCult-XF medium (STEMCELL Technologies Inc., Canada). The mesenchymal tissues in umbilical cord Wharton’s jelly were diced into cubes of approximately 0.5 cm^3^ and washed with Hanks’ balanced saline solution (Gibco Invitrogen). Then, the tissue pellets were seeded in a tissue culture flask (Corning Enterprises, Corning, NY) in α-MEM supplemented with 10% fetal bovine serum (STEMCELL Technologies, Vancouver, BC, Canada). The medium was replenished every 3 days. The UC-MSC was cultured and collected between the third and fourth passages for infusion.

For quality control of the UC-MSC, the cultured cells at the third passage were examined for the expression of their phenotypes by flow cytometric analysis, for example, high expression of CD44, CD73, CD90, and CD105, but no expression of CD31, CD34, CD45, or HLA-DR. Alternatively, the digested cells were cultured in conditioned medium (STEMCELL Technologies, Vancouver, BC, Canada), and were subsequently cultured for osteogenesis and adipogenesis differentiation assays. Briefly, the digested cells were cultured in osteogenesis-inducing medium (Gibco Invitrogen) consisting of MesenCult basal medium, osteogenic stimulatory supplements, dexamethasone, *β*-glycerophosphate and ascorbic acid ion for 5 weeks, or in adipogenic induction medium (Gibco Invitrogen) consisting of MesenCult basal medium and MesenCult adipogenic stimulatory supplements for 2 weeks. Subsequently, osteogenesis was assessed by alkaline phosphatase staining, and adipogenesis was assessed by oil red O staining. Moreover, the UC-MSC was negative for all the tested contaminants before infusion, including *Mycoplasma* spp., gram-positive and gram-negative bacteria, and fungi. The endotoxin levels were below 5 EU/kg and viability was > 80%.

Patients in the control group received the conventional treatment which included on targeting abnormalities in gut-liver axis by antibiotic administration (i.e., rifaximin), improving the disturbed systemic circulatory function (i.e., long-term albumin administration), decreasing the inflammatory state (i.e., statins), and reducing portal hypertension (i.e., beta-blockers) be used to decrease cirrhosis progression in patients with decompensated cirrhosis. Management of specific complications of decompensated cirrhosis is followed by the practice guidelines for each complications of decompensated cirrhosis (such as variceal bleeding, ascites and encephalopathy).

**Fig. 1:**
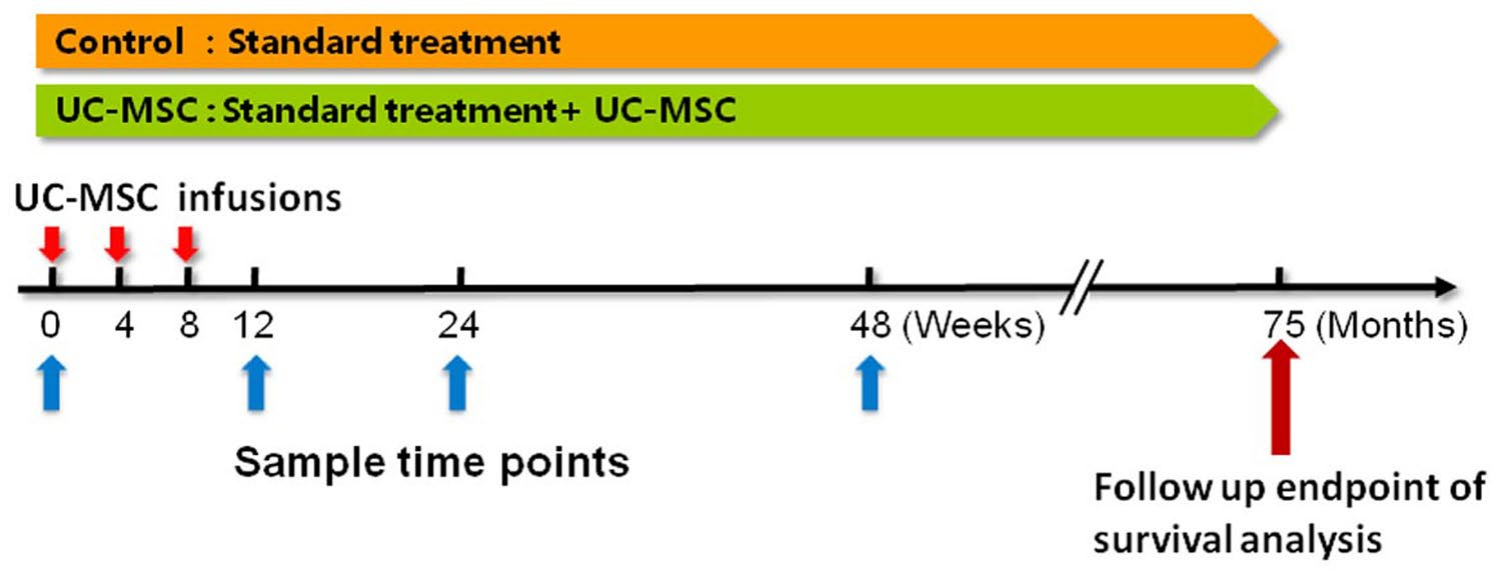
Protocol for UC-MSC treatment in HBV-related decompensated liver cirrhosis patients. UC-MSC transfusions were administered to the patients three times: at the baseline (0 weeks), at 4 weeks, and at 8 weeks. The clinical parameters were tested at 0, 12, 24, and 48 weeks after the treatment. For survival analysis, the follow-up was prolonged to 75 months. All participants received the same conventional treatment throughout the study

In the UC-MSC treatment group, patients received UC-MSC therapy plus the conventional treatment. The UC-MSC therapy was administrated at a dose of approximately 0.5 × 10^6^/kg body weight UC-MSC at the fourth passage, which were suspended in saline and were infused intravenously three times at 4-week intervals (Fig. 1). The patients in the UC-MSC treatment group were observed for 6 h after the UC-MSC therapy before discharged from the clinic.

After first UC-MSC infusion, the patients were followed up at weeks 2, 4, 8, 12, 24, and 48, and at months 24, 48, 60, and 75. The following tests were performed at weeks 0, 12, 24, and 48: liver function tests: serum ALB, prothrombin activity (PTA), cholinesterase (CHE) and TBIL. The adverse events were collected during 48 weeks after the first infusion of UC-MSC. The adverse events and their severity were determined by means of vital signs and physical examinations, laboratory examinations, oral and telephone inquiries.

### Outcome measures

The primary outcome measures were overall survival rate and the hepatocellular carcinoma (HCC)-free survival rate after UC-MSC infusions in each treatment group. The secondary outcome measures included the incidence of adverse events (e.g., fever, allergy, rash, and infection), effects of the treatment on liver function (including the levels of albumin [ALB], prothrombin activity [PTA], TBIL, and CHE), and the incidence of serious complications (including infection, gastrointestinal bleeding, encephalopathy, and hepatorenal syndrome).

### Statistical analysis and sample size

The median overall survival was assumed to be 60 months in 60% of the patients who received the conventional medical treatment, and in 85% of those who received the UC-MSC therapy. We planned to recruit 100 patients in each group during a recruitment period of 40 months and follow for over 75 months, which would have more than 80% power to detect a significant risk difference between the two groups, at a two-sided *α* level of 0.05 (PASS 11.0). Based on the dropout or withdrawal rate, we determined a sample size of 125 patients per group.

All eligible participants who received the assigned treatment and followed up were included in the analysis. We counted the number of endpoint events (death or HCC events) that occurred during the follow-up period and compared the event-free survival rate between the two groups using Kaplan–Meier analysis. The post-treatment follow-up was continued until the date of an endpoint event, death, or the end of the study, whichever occurred first. Cox proportional-hazards models were developed to estimate the hazard ratio (HR) and 95% confidence interval (CI) for between-group comparisons with or without adjustments for stratification baseline model of end-stage liver disease (MELD) scores. In addition, we performed landmark analyses to assess the endpoint events accordingly, with the hazard ratio calculated separately for events that occurred before or after the landmark points. Hypothesis testing was two-sided with an *α* value of 0.05. The χ^2^ test or Fisher’s exact test was used to analyze categorical data, while the Student’s *t* test or paired Student’s *t* test was used to analyze continuous data.

## Results

### Patients and enrollment

**Fig. 2:**
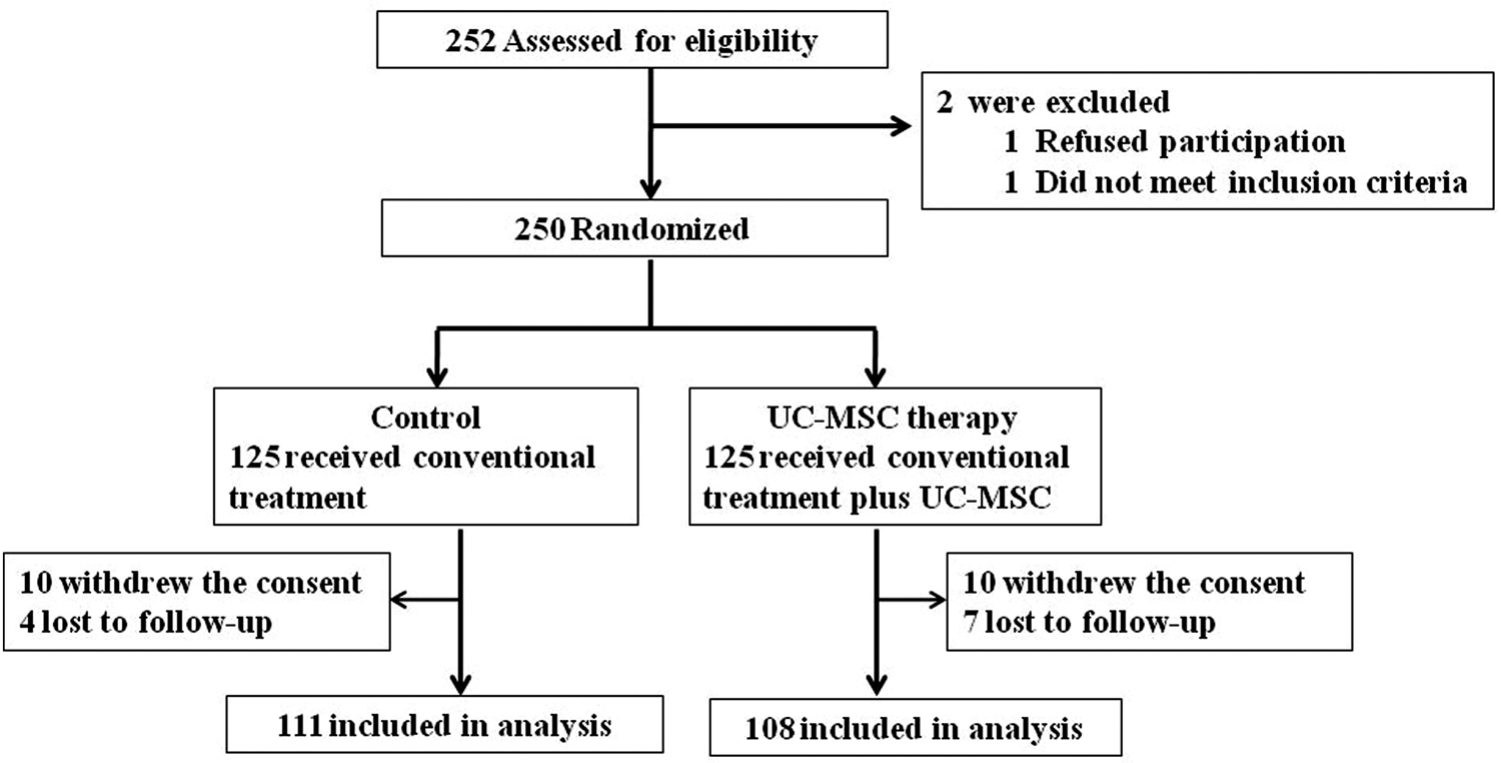
Study flow diagram. A total of 252 patients with decompensated liver cirrhosis were enrolled, but two patients were excluded: one refused to participate, and one did not meet the inclusion criteria. Therefore, 250 patients were randomized into the control and umbilical cord-derived mesenchymal stem cell (UC-MSC) treatment groups, with 125 patients in each group. In the control group, ten patients withdrew the consent, four patients were lost to follow-up, and in the UC-MSC treatment group, ten patients withdrew the consent, seven patients were lost to follow-up. Therefore, 111 control patients and 108 UC-MSC-treated patients were followed up for 75 months and analyzed in the trial

A total of 252 decompensated liver cirrhosis patients who had chronic HBV infection were recruited and screened between October 15, 2010 and October 30, 2017. Among them, two patients were excluded because they refused to participate (*n* = 1), or failed to meet the inclusion criteria (*n* = 1). Therefore, 250 patients were randomly assigned to the control and UC-MSC treatment groups (*n* = 125 in each group). In the control group and UC-MSC treatment group, 10 and 10 patients withdrew the consent, and 4 and 7 patients were lost to follow-up, respectively. Therefore, 111 control patients and 108 UC-MSC treated patients were included in the primary analysis and followed up for 75 months (Fig. 2). The median follow-up of patients was 61 months in UC-MSC treatment group (range 1–85 months), and 57 months in control group (range from 2 to 82 months).

The baseline clinical parameters of the patients in the two groups are shown in Table 1. The baseline levels of total bilirubin (TBIL) (*p* < 0.001) and MELD score (*p* = 0.040) in the UC-MSC treatment group were higher than those in the control group; in addition, the baseline level of cholinesterase (CHE) in the UC-MSC treatment group was lower than that in the control group (*p* = 0.008). The other parameters at the baseline were comparable between the two groups. The patients in the UC-MSC treatment group were administered UC-MSC infusions, whereas the patients in the control group were administered the same volume of saline. All participants received the same conventional treatment throughout the study; for example, all the CHB patients received antiviral therapy with entecavir (0.5 mg daily), and the serum HBV DNA levels were below detection limit level (< 100 IU/mL) when enrolled in this study. In addition, patients with ascites in both the UC-MSC and control groups received the same dose of diuretics (spironolactone [40 mg daily] plus furosemide [20 mg daily]) until the ascites was treated. Other medications that might affect the outcome were not given in this study.

**Table 1:** Baseline characteristics of the patients

	Control (*n* = 111)	UC-MSC (*n* = 108)	*p* value
Age (years)			
Median	47.0	48.0	
Range	19–65	21–65	0.411
Gender (male/female)	96/15	94/14	0.904
TP (g/L)	61.5 ± 7.4	62.8 ± 8.0	0.209
ALB (g/L)	30.5 ± 6.2	30.9 ± 5.9	0.637
PLT (cells/mm^3^)	83 ± 95	90 ± 105	0.608
Cr (μmol/L)	84.9 ± 62.6	82.5 ± 23.4	0.709
ALT (U/L)	59.6 ± 79.4	42.4 ± 49.16	0.058
AST (U/L)	70.4 ± 61.5	65.8 ± 43.7	0.521
TBil (μmol/L)	33.2 ± 29.8	76.0 ± 127.9	< 0.001
PTA (%)	62.4 ± 16.3	63.3 ± 16.5	0.691
CHE (U/L)	3337 ± 1335	2812 ± 1560	0.008
INR	1.32 ± 0.21	1.33 ± 0.26	0.777
HBeAg-positive (%)	28 (25.2%)	30 (27.8%)	0.669
MELD score	10.0 ± 4.2	11.4 ± 6.0	0.040
> 15, % (*n*/total number)	13.5 (15/111)	17.6 (19/108)	
< 15, % (*n*/total number)	86.5 (96/111)	82.4 (89/108)	0.405
Child–Pugh score (*n*)			
A	20	14	0.266
B	73	68	
C	18	26	
Ascites (presence/absence)	93/18	86/22	0.426

Data are expressed as the mean ± standard deviation

*ALB* albumin, *ALT* alanine aminotransferase, *AST* aspartate aminotransferase, *CHE* cholinesterase, *Cr* creatinine, *INR* international normalized ratio, *MELD score* model of end-stage liver disease score, *PLT* platelet, *PTA* prothrombin activity, *TBil* total bilirubin, *TP* total protein

### Effects of UC-MSC on long-term survival

**Fig. 3:**
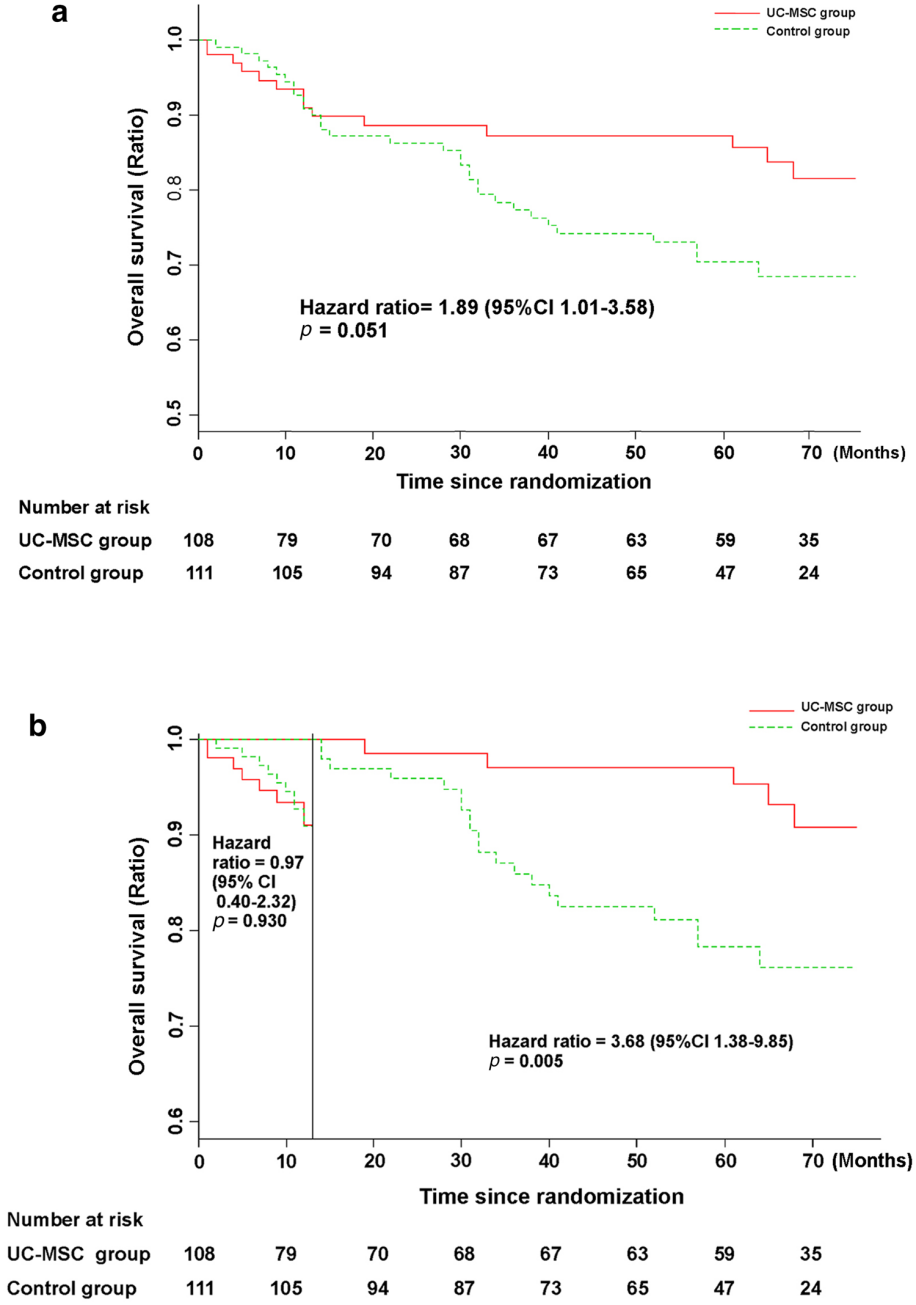
Disease-free survival rate in patients with HBV-related decompensated liver cirrhosis treated with UC-MSC infusion or the conventional therapeutic regimen. **a** Disease-free survival rate in the UC-MSC treatment group and control group. **b** Landmark analysis for comparing survival before 13 months and beyond 13 months (up to 75 months) of follow-up

At the 75-month follow-up, the overall survival rate did not differ significantly between the UC-MSC treatment group and control group (Fig. 3a) (HR = 1.89, 95% CI 1.01–3.58, *p* = 0.051). While using Landmark analysis, the overall survival rate was significantly higher in the UC-MSC treatment group than in the control group during the 13- to 75-month follow-up (Fig. 3b) (HR = 3.68, 95% CI 1.38–9.85, *p* = 0.005), although the overall survival rate did not differ significantly between the UC-MSC treatment group and control group within the first 13-month follow-up (HR = 0.97, 95% CI 0.40–2.32, *p* = 0.930). These results indicate that although the UC-MSC treatment can significantly increase the survival rate in patients with decompensated liver cirrhosis, the superiority of the UC-MSC treatment to the conventional medical treatment may be evident only about 13 months after UC-MSC infusion.

### Effect of UC-MSC infusions on liver functions

**Fig. 4:**
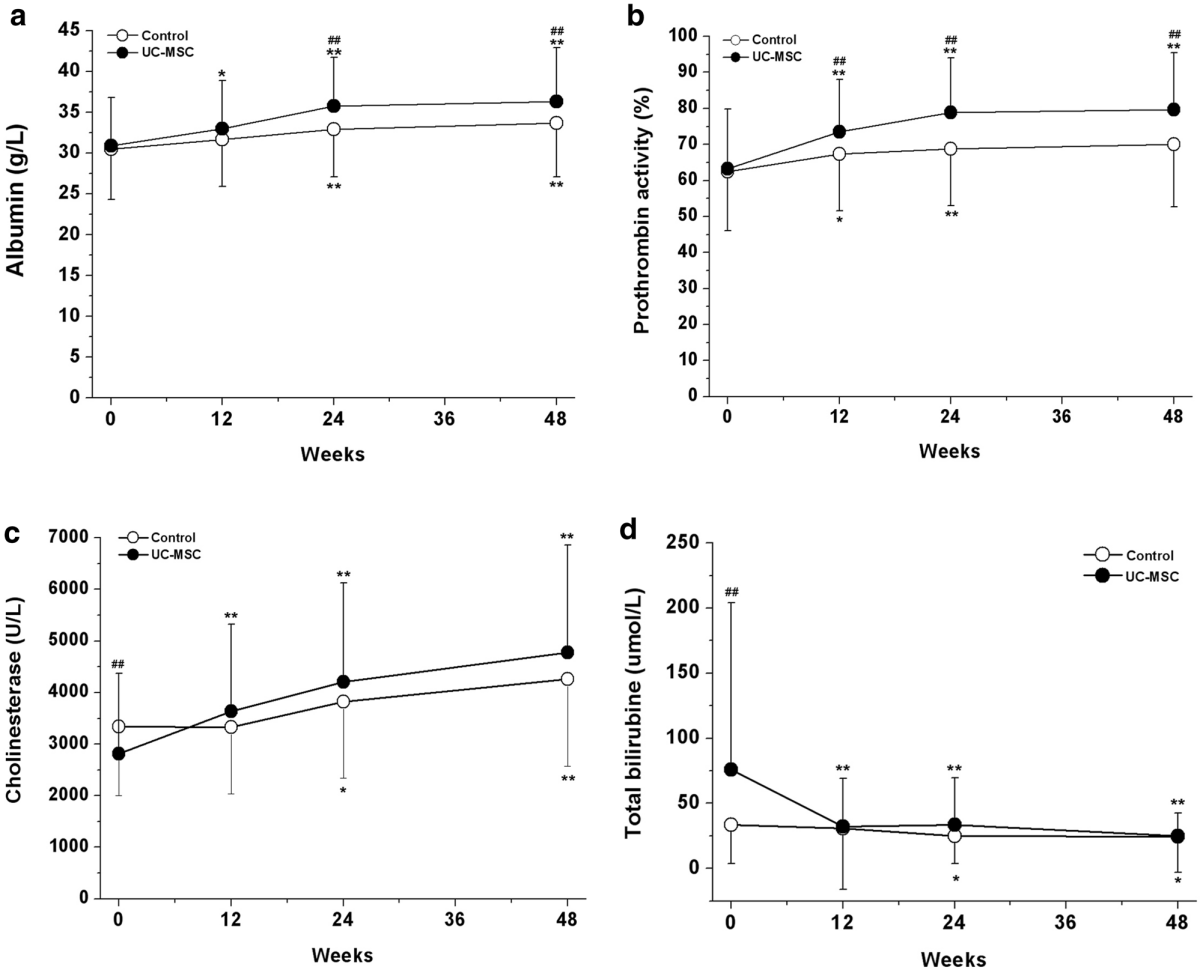
Influence of UC-MSC treatment on liver function in decompensated liver cirrhosis patients. **a** Serum albumin, **b** prothrombin activity, **c** cholinesterase, **d** total bilirubin. *p < 0.05, **p < 0.01 compared with the baseline values; ^##^*p* < 0.01 compared with the control group at the corresponding time point

To investigate the impact of UC-MSC infusion on liver function, the serum levels of ALB, PTA, CHE and TBIL were monitored within 48 weeks after the first UC-MSC infusion or conventional medical treatment. The levels of ALB had increased in comparison with the baseline in both groups during the 48-week follow-up, but it was significantly higher in the UC-MSC treatment group at the 24- and 48-week time points (Fig. 4a). The levels of PTA in the UC-MSC treatment group were markedly increased as compared with the baseline and control group during the 48-week follow-up (Fig. 4b). Astonishingly, the baseline level of CHE in the UC-MSC treatment group was obviously lower and the level of TBIL was obviously higher than that in the control group. After the UC-MSC infusions, the level of CHE was obviously increased and the level of TBIL was markedly decreased as compared with the baseline level during the 48-week follow-up; however, there was no significant difference in comparison with the control group (Fig. 4c, d). These data indicate that the UC-MSC therapy could alleviate hepatoinflammation and obviously improve liver function.

### Adverse effects and safety of UC-MSC infusions

**Fig. 5:**
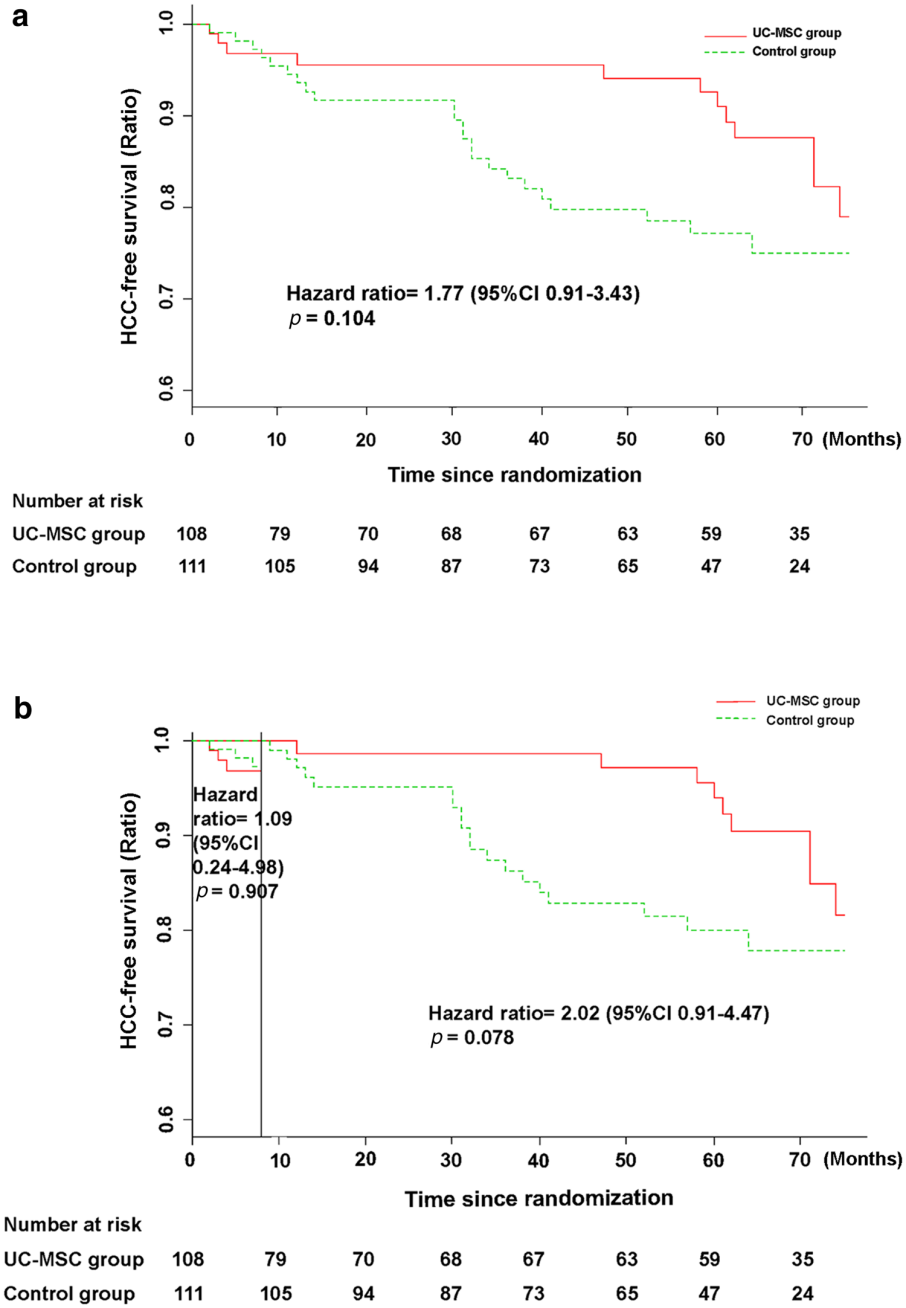
HCC event-free rate in patients with HBV-related decompensated liver cirrhosis treated with UC-MSC infusion or the conventional treatment protocol. **a** HCC event-free rate in the UC-MSC treatment group and control group. **b** Landmark analysis for comparing survival before 8 months and during 8–75 months of follow-up

All the patients in the UC-MSC group tolerated the UC-MSC treatment well. Seven patients developed a self-limiting fever (body temperature, 37–38 °C) within 2–6 h after the UC-MSC transfusions but recovered within 12 h without any additional treatment. No other short-term clinical adverse effects, including allergy, rash, or infection were found. Long-term safety is a major concern in UC-MSC therapy for these patients. Therefore, we monitored the long-term adverse events, such as HCC events, during the 75-month follow-up after the first UC-MSC infusion. At the 75-month follow-up, cumulative incidences of HCC in control group and MSC treatment group were 20.7% (23/111) and 12.0% (13/108), respectively. However, the Kaplan–Meier analysis showed that there was no significant difference of HCC-free survival rate between the UC-MSC treatment group and control group (HR = 1.77, 95% CI 0.91–3.43, *p* = 0.104) (Fig. 5a). Interestingly, the HCC disease-free survival rate was seemingly higher in the control group than in the UC-MSC treatment group within the first 8 months of follow-up, but it was higher in the UC-MSC group between 8 and 75 months. However, landmark analysis did not reveal any significant difference in the HCC disease-free survival rate between the UC-MSC treatment group and control group within 8 months (HR = 1.09, 95% CI 0.24–4.98, *p* = 0.907) or between 8 and 75 months (HR = 2.02, 95% CI 0.91–4.47, *p* = 0.078) (Fig. 5b). These results indicate that the UC-MSC treatment did not pose a higher risk than the conventional treatment in terms of long-term safety for patients with decompensated liver cirrhosis.

## Discussion

In the present study, we monitored patients treated with UC-MSC for the longest follow-up period reported to date, that is, 75 months, to understand the longer term effects of UC-MSC treatment in chronic hepatitis B-related decompensated liver cirrhosis. Apart from the benefits of the treatment with regard to improving liver function and survival, no significant adverse events or undesired effects were observed for up to 75 months of follow-up. This finding is important, as safety is a major concern in the clinical application of UC-MSC. Thus, our data revealed that UC-MSC treatment is safe and does not increase the occurrence of HCC events, and it has a long-term effect on improving liver function and survival in patients with decompensated liver cirrhosis.

MSC have been found to be chemoattracted toward the tumor microenvironment, where they exert controversial effects as supporters or inhibitors of tumor progression [16–25]. Fortunately, human MSC seem to be at a lower risk of malignant transformation, as they can be safely expanded at the 15th passage in vitro and are not susceptible to malignant transformation in serum-free medium [26]. The safety results of a pool of 700 subjects who received autologous or third-party MSC showed that none of them had major side effects or induced the development of hematopoietic or solid tumors [27]. In our clinical trial, to guarantee the safety of patients undergoing UC-MSC treatment, the UC-MSC was cultured in serum-free medium and collected between the third and fourth passages for application, and patients who showed any detectable tumor were excluded. In fact, the landmark analyses in our clinical trial showed that the HCC disease-free survival rate was not significantly different between the UC-MSC treatment group and the control group even at the 75-month follow-up. Thus, our findings confirm the long-term safety of this treatment for decompensated liver cirrhosis.

In the present study, UC-MSC treatment was associated with improvement in liver function, as indicated by the enhanced ALB, PTA and CHE levels and decreased TBIL level, during the 48-week follow-up. However, with regard to survival, there was no obvious difference between the UC-MSC treatment group and the control group within the 13-month follow-up, and the overall survival rate was significantly higher in the UC-MSC treatment group only during the 13–75 months follow-up. This means that the efficacy of UC-MSC treatment for decompensated liver cirrhosis is obvious only after 13 months. The survival benefit might be associated with improvement in liver function and partial blockage or reversal of liver cirrhosis, through various mechanisms that have been reported before. For example, UC-MSC is known to home into the injured liver and differentiate into hepatocyte-like cells in vivo [28], facilitate resident hepatocyte proliferation via paracrine activity by secreting high levels of hepatic growth factor [12, 29], and play an immunomodulatory role in reducing inflammation by producing inhibitory cytokines or inducing the development of regulatory T cells [30–32]. However, the exact mechanism by which UC-MSC treatment improves survival only after 13 months of UC-MSC treatment remains to be further investigated.

Several limitations were also present in this study. First, we did not document the histological alterations in the liver in the studied patients because liver biopsy examination was a high-risk operation in the patients with decompensated liver cirrhosis. Second, we did not track the infused UC-MSC in patients in vivo due to technical and ethical issues. Third, immunoregulation functions of MSCs more likely contribute to decrease the inflammation cytokines production in DLC patients, the associated cytokines have not been conducted. Fourth, this is a single-center clinical trial, which may have a selective bias in the enrollment of cases. Thus, multi-center clinical trials should be conducted to confirm the use of UC-MSC treatment in such patients in further studies.

In summary, UC-MSC infusion is safe and tolerable, and can improve survival in patients with HBV-related decompensated liver cirrhosis, which is expected to be generalizing and applied in clinical practice and become a novel therapeutic strategy for the treatment of HBV-related decompensated liver cirrhosis.

## Supplementary Information

Below is the link to the electronic supplementary material.

Supplementary file1 (PDF 236 kb)

Supplementary file2 (PDF 510 kb)

## Data Availability

All the data generated and analyzed during this study are included in our manuscript. The data that support the findings of this study are available from the corresponding author upon reasonable request.
