# Supplementary material for: Mesenchymal stem cell therapy in decompensated liver cirrhosis: a long-term follow-up analysis of the randomized controlled clinical trial
Source: Hepatol Int. 2021 Nov 29;15(6):1431–41. doi: 10.1007/s12072-021-10199-2 (PMC8651584; doi:10.1007/s12072-021-10199-2)
Supplement: Supplementary file 1 — Supplementary file1 (PDF 236 kb) [file 12072_2021_10199_MOESM1_ESM.pdf]

CLINICAL PROTOCOL

Human Umbilical Cord Mesenchymal Stem Cells to Treat Decompensated Liver Cirrhosis

Objective: To evaluate the safety and efficacy of human umbilical cord mesenchymal stem cells in the treatment of decompensated liver cirrhosis

Clinical research sponsor: The Treatment and Research Center for Infectious Diseases, Beijing 302 Hospital of PLA

Principal Investigator: Fu-Sheng WANG

Unit responsible for clinical research: The Treatment and Research Center for Infectious Diseases, Beijing 302 Hospital of PLA

Protocol Version Number: V 2.0

Registration Number: NCT01220492

Revision date: October 12, 2010

SYNOPSIS

Title	Human Umbilical Cord Mesenchymal Stem Cells to Treat Decompensated Liver Cirrhosis
Registration Number	NCT01220492
Indication	Decompensated liver cirrhosis associated with chronic hepatitis B
Objectives	Primary objective: To evaluate the efficacy of human umbilical cord mesenchymal stem cells to treat decompensated liver cirrhosis.<br>Secondary objective: To evaluate the safety of human umbilical cord mesenchymal stem cells in the treatment of decompensated liver cirrhosis.
Unit responsible for clinical research	Beijing 302 Hospital of PLA
Clinical Trial Type	Initiated by researchers
Participants	Patients with decompensated liver cirrhosis associated with Chronic Hepatitis B
Sample Size	250 cases
Study Design	Prospective, single center, open label, randomized controlled trial
Investigational Agents	Umbilical cord mesenchymal stem cells (UC-MSC) derived from healthy fetal umbilical cords and processed by pulverization, centrifugation, culture, and activation *in vitro*.
Administration Regimen	Treatment group: Conventional treatment + UC-MSC treatment, 0.5×10^6 cells/kg body weight, infused intravenously three times at 4-week intervals.<br>Control group: Conventional treatment according to Chronic Hepatitis B Prevention and Treatment Guidelines (APASL 2008), Cirrhotic Ascites Prevention and Treatment Guidelines (AASLD 2004), and Cirrhotic Variceal Hemorrhage Prevention and Treatment Guidelines (AASLD 2008).
Inclusion Criteria	(1) Patients aged 18 to 65 years.<br>(2) Patients testing positive for serum hepatitis B surface antigen (HBsAg) for more than 6 months.<br>(3) Diagnosis of decompensated liver cirrhosis, which was defined as liver cirrhosis with serious complications, such as ascites, hepatic encephalopathy, and esophageal and gastric variceal hemorrhage according to the Prevention and Treatment of Chronic Hepatitis B (China 2009).<br>(4) Negative pregnancy test (female patients of fertile age).<br>(5) Willing to participate in this clinical trial, and able to understand and

	provide informed consent.
Exclusion Criteria	(1) Hepatocellular carcinoma or other malignancies.<br>(2) Pregnancy or lactation.<br>(3) Sepsis.<br>(4) Presence of significant extrahepatic biliary disease (e.g., common bile duct (CBD) stone and primary sclerosing cholangitis (PSC)).<br>(5) Severe renal, cardiac, or respiratory disease.<br>(6) Active thrombosis of the portal or hepatic veins.
Withdrawal criteria	(1) The participant's request to stop test for lack of efficacy, intolerance, or adverse reactions.<br>(2) The participant's compliance was poor, and they could not infuse stem cells as required or follow the protocol.<br>(3) The female participants were pregnant or lactating during the clinical trial.<br>(4) Any clinical adverse event (AE), laboratory abnormality or intercurrent illness which, in the opinion of the investigator, indicates that continued participation in the study is not in the best interest for the subject.<br>(5) Other conditions unsuitable to continue test in the opinion of the investigator.
Termination Criteria	(1) Serious safety problems occurred during the test.<br>(2) Termination requested by clinical research sponsor (such as funding or management reasons).<br>(3) Termination requested by independent ethics committee or administrative department.
Visit Schedule	Treatment times: Week 0, week 4, week 8.<br>Follow-up periods: Week 2, week 4, week 8, week 12, week 24, week 48, month 24, month 48, month 60, month 75.
Endpoints	Primary endpoints<br>(1) Overall survival rate during 75 months posttreatment follow up.<br>(2) Hepatocellular carcinoma (HCC)-free survival rate during 75 months posttreatment follow up.<br>Secondary endpoints<br>(1) Liver function including the levels of albumin (ALB), prothrombin activity (PTA), total bilirubin (TBIL), and cholinesterase (CHE).<br>(2) Complications including gastrointestinal hemorrhage, abdominal infection, hepatic encephalopathy, and hepatorenal syndrome during 48 weeks after the first UC-MSC infusion.

VISIT SCHEDULE

Visit item	Screening period	On treatment & Posttreatment visit period											
		Treatment 1	Visit 1	Treatment 2	Treatment 3	Visit 2	Visit 3	Visit 4	Visit 5	Visit 6	Visit 7	Visit 8
Visit time	−7~0 Day	Week 0	Week 2 ±2 days	Week 4 ±2 days	Week 8 ±7 days	Week 12 ±7 days	Week 24 ±14 days	Week 48 ±14 days	Month 24 ±15 days	Month 48 ±15 days	Month 60 ±30 days	Month 75 ±30 days
Informed consent	×											
Demographics	×											
Medical history	×											
Weight	×	×	×	×	×	×	×	×				
Vital signs	×	×	×	×	×	×	×	×				
Physical examination	×	×	×	×	×	×	×	×				
Urine pregnancy test (female)	×		×		×	×	×					
Routine blood test	×		×	×	×	×	×	×				
Routine urine test	×		×	×	×	×	×	×				
Biochemical test	×		×	×	×	×	×	×				
Coagulation function	×		×	×	×	×	×	×				

Visit item	Screening period	On treatment & Posttreatment visit period										
		Treatment 1	Visit 1	Treatment 2	Treatment 3	Visit 2	Visit 3	Visit 4	Visit 5	Visit 6	Visit 7	Visit 8
Visit time	−7~0 Day	Week 0	Week 2 ±2 days	Week 4 ±2 days	Week 8 ±7 days	Week 12 ±7 days	Week 24 ±14 days	Week 48 ±14 days	Month 24 ±15 days	Month 48 ±15 days	Month 60 ±30 days	Month 75 ±30 days
HbA1c	×											
Five items of hepatitis B	×											
HBV DNA	×					×	×	×				
AFP	×					×	×	×				
Anti-HCV	×											
Anti-HIV	×											
Abdominal ultrasound	×					×	×	×				
Chest X-ray	×											
Electrocardiogram (ECG)	×											
Adverse events (AEs)			×	×	×	×	×	×				
Complications			×	×	×	×	×	×				
UC-MSC infusion		×		×	×							

Visit item	Screening period	On treatment & Posttreatment visit period										
		Treatment 1	Visit 1	Treatment 2	Treatment 3	Visit 2	Visit 3	Visit 4	Visit 5	Visit 6	Visit 7	Visit 8
Visit time	−7~0 Day	Week 0	Week 2 ±2 days	Week 4 ±2 days	Week 8 ±7 days	Week 12 ±7 days	Week 24 ±14 days	Week 48 ±14 days	Month 24 ±15 days	Month 48 ±15 days	Month 60 ±30 days	Month 75 ±30 days
Survival status (follow-up by telephone)									×	×	×	×
Occurrence of HCC (follow-up by telephone)									×	×	×	×

Abbreviation

**ACLF**	**acute-on-chronic liver failure**
**ADL**	**activities of daily living**
**AE**	**adverse events**
**AFP**	**alpha-fetoprotein**
**ALB**	**albumin**
**ALT**	**alanine aminotransferase**
**AST**	**aspartate aminotransferase**
**CHE**	**cholinesterase**
**CLcr**	**creatinine clearance rate**
**Cr**	**creatinine**
**CRF**	**case report form**
**ECM**	**extracellular matrix**
**FAS**	**full analysis set**
**FDA**	**Food and Drug Administration**
**GCP**	**Good Clinical Practice**
**GMP**	**Good Manufacturing Practice**
**Hb**	**hemoglobin**
**HbA1c**	**glycosylated hemoglobin**
**HBsAg**	**hepatitis B surface antigen**
**HBV**	**hepatitis B virus**
**HCC**	**hepatocellular carcinoma**
**HCV**	**hepatitis C virus**
**HGF**	**hepatocyte growth factor**
**HIV**	**human immunodeficiency virus**

**HSC**	**hepatic stellate cells**
**IL**	**interleukin**
**INR**	**international standardization ratio**
**MedDRA**	**Medical Dictionary for Regulatory Activities**
**MELD**	**model of end-stage liver disease**
**MSC**	**mesenchymal stem cells**
**NK**	**natural killer cells**
**NYHA**	**New York Heart Association**
**PLT**	**platelet**
**PPS**	**per protocol set**
**PT**	**prothrombin time**
**PTA**	**prothrombin activity**
**SAE**	**serious adverse event**
**SS**	**safety set**
**TBIL**	**total bilirubin**
**TEAE**	**treatment emergent adverse event**
**TGF**	**transforming growth factor**
**TNF**	**tumor necrosis factor**
**Treg**	**regulatory T cell**
**UC-MSC**	**umbilical cord mesenchymal stem cells**
**ULN**	**upper limit of normal**
**WBC**	**white blood cells**

CATALOGUE

1 Background……………………………………………………………………………..1

2 Objectives………………………………………………………………………….…2

3 Study Design……………………………………………………………………….2

3.1 Overall design and sample size………………………………………………………2

3.2 Endpoints………………………………………………………………………….3

3.2.1 Primary endpoints……………………………..…………………………….3

3.2.2 Secondary endpoints…………………………..…………………………….3

3.3 Randomization……………………..…………………..………………………….3

4 Participants……………………..……………………..……..……………..……………4

4.1 Inclusion Criteria………..………………………..……...……………..………………4

4.2 Exclusion Criteria………..………………………......………………..………………...4

4.3 Withdrawal criteria……..………………………......………………..……………..…...5

4.4 Termination criteria…..………………………......………………..………...……..…...6

5 Interventions……………………..……………………..……..…………….……….…….6

5.1 Human mesenchymal stem cells……………………......………………..………...……...6

5.2 Conditions for preparation and monitoring……….......………………..………...……...7

5.2.1 Umbilical cord collection……………………......………………..………..……7

5.2.2 UC-MSC quality control……………………......………………..………...……9

5.3 Used dosage of UC-MSC………………….....……………………….……….....……9

5.4 Treatment method and course of treatment…......……………………..…………….……9

5.5 Conventional clinical treatment…………….....……………………...…..………...…10

6 Research process……………………..……………………..……..……………….……..…10

6.1 Follow-up process…………………......……………………..…..………..…..……11

6.2 Screening period(−7~0 days) ………………......……………………………………..14

6.3 Treatment 1 (week 0) baseline phase………….....……………………………………20

6.4 Visit 1 (2nd week ±2 days) ……….……….....……………………………………20

6.5 Treatment 2 (4th week ± 2 days) ……………………………………………………………20

6.6 Treatment 3 (8th week ± 7 days) ……………………………………………………………21

6.7 Visit 2 (12th week ± 7 days) ………………………………………………………………21

6.8 Visit 3 (24th week ± 14 days) ……………………………………………………………..21

6.9 Visit 4 (48th week ± 14 days) ……………………………………………………………...22

6.10 Visit 5 (24 months ± 15 days) ……………………………………………………………22

6.11 Visit 6 (48 months ±15 days) ……………………………………………………………..23

6.12 Visit 7 (60 months ±30 days) ……………………………………………………………23

6.13 Visit 8 (75 months ± 30 days) ……………………………………………………………23

6.14 Unplanned visits (when the study requires it or after withdrawal from the trial ahead of time) …………………………………………………………………………………………23

7 Efficacy Assessments…………………………………………………………………………23

7.1 Survival analysis…………………………………………………………………………23

7.2 Secondary Efficacy Assessments…………………………………………………………23

8 Concomitant Diseases and Treatments……………………………………………………24

8.1 Concomitant diseases…………………………………………………………………24

8.2 Concomitant treatments…………………………………………………………………24

9 Safety evaluation……………………………………………………………………………24

9.1 Definition of AEs………………………………………………………………………25

9.2 Definition of SAE……………………………………………………………………...25

9.3 Abnormal clinical test or other abnormal indicators as adverse events or serious adverse events……………………………………………………………………………………...26

9.4 AE and SAE Collection and Recording…………………………………………………...27

9.5 AE and SAE assessment……………………………………………………………………...27

9.5.1 Severity determination of an AE………………………………………………...27

9.5.2 The judgment of causality…………………………………………………………...28

9.6 AE and SAE follow-up…………………………………………………………………...28

9.6.1 SAE Reporting……………………………………………………………………...29

9.6.2 AEs and SAEs after the study……….…………………………….……….…..…...30

9.6.3 AE and SAE related to participation in the trial………………….……….…..…...30

10 Withdrawal from the study………..………………………………….……….……..…...30

10.1 Subjects accomplish the trail…………………………………..……….………..…...30

10.2 Subjects withdraw from the trial………………………………….…………….…..…...30

11 Data Management…………………………………………………….…………..…...31

12 Statistical analysis…………………………………………..………………..…...31

12.1 Sample size……….…………………………………..……………………..…...31

12.2 Definition and selection of analysis sets……………………..………….………..…...31

12.3 Statistical methods…….……………………………..……………….…………….32

12.3.1 Case group analysis…………………………..……………..…………32

12.3.2 Demographic data and baseline analysis………….……………..…………32

12.3.3 Safety Analysis……………………………………..……………………32

12.3.4 Efficacy analysis…………………..……………………………………33

12.4 Statistical software and general requirements………………………..….……………34

13 Clinical trial management………………………………………………….….……………34

13.1 Compliance with GCP requirements……………………………..…...…..……………34

13.2 Protecting the privacy of the subject……………………………..…...…..……………34

13.3 Informed consent form……………………..……………………..…...…..……………34

13.4 Program revision procedures…………………..…………………...…………………35

13.5 Study medical record and case report form record…………………...…………………35

13.6 Quality control and quality assurance……………………….....……………………35

13.7 Data storage……………………………………………………...……………………36

14 Risks and benefits………………………………………………..…..……….……………36

15 Papers published………………………..……………………………..…..……….……………36

16 Ethics…………………………..………..……………………..…..…..……….……………37

17 References………………………..………..……………………..…..…..……….……………38

1 Background

Liver cirrhosis is one of the late stages of progressive hepatic fibrosis, which is characterized by the formation and accumulation of an extracellular matrix, and leads to the progressive distortion of the hepatic architecture [1]. In China, the most important cause of liver cirrhosis is chronic hepatitis B virus (HBV) infection. Liver cirrhosis usually progresses irreversibly into the advanced stage, for example, the decompensated stage, which is characterized by a series of clinical manifestations, including ascites, variceal hemorrhage, and hepatic encephalopathy, with high mortality. Liver transplantation is the only treatment that can improve the survival of patients with decompensated liver cirrhosis; however, this procedure is associated with several limitations, such as the severe shortage of donor livers, long waiting lists, multiple complications, and high cost. Therefore, there is an urgent need to find an alternative therapeutic approach to treat decompensated liver cirrhosis that is both safe and effective.

Stem cells, including embryonic stem cells and adult stem cells, have strong practicality and favorable clinical prospects. Mesenchymal stem cells (MSC), a kind of adult stem cell, are multipotent stem cells derived from mesenchymal cells, which have unlimited self-renewal ability and the potential to differentiate into specific cell lineages, including hepatocytes. MSC have been reported to be successfully isolated from different tissues with the common characteristics, including bone marrow [2], umbilical cord blood [3], adipose tissue [4], and the placenta [5]. Umbilical cord tissue is an especially promising source of MSC. All umbilical veins, umbilical arteries, sub-amniotic tissues, Wharton's jelly, and umbilical cord perivascular tissues could be sources of MSC. Three criteria have been defined by the International Society for the isolation and purification of MSC for cellular therapy: 1) Adherence to plastic under standard culture conditions; 2) expression of CD105, CD73, and CD90; 3) lack of expression of hematopoietic and endothelial markers including CD11b, CD14, CD31, CD34, CD45, and HLA-DR; differentiation into adipocytes, osteocytes, and chondrocytes *in vitro* under specific culture conditions [6]. In the past decade, MSC therapies have emerged as a novel alternative to treat end-stage liver diseases.

The potential for stem cells to differentiate into hepatocytes cells was confirmed recently. In particular, bone marrow derived mesenchymal stem cells (BM-MSC) transplantation has been applied in the clinic to treat several human diseases such as graft-versus-host disease (GVHD), cardiac injury and brain injury, displaying good tolerance and efficiency [7]. BM-MSC have also been used to treat human liver diseases, such as liver failure and liver cirrhosis. In a phase 1 study, autologous BM-MSC transplantation showed potential to decrease the MELD score and increase serum albumin in patients with decompensated liver cirrhosis [8, 9].

MSC can exert anti-fibrotic effects directly or indirectly by inhibiting the proliferation of hepatic stellate cells (HSC), extracellular matrix (ECM) deposition and inducing HSC apoptosis. HSC, the leading mediators of liver fibrosis, deposit the ECM under conditions of liver injury, pathological factors and cytokines, eventually leading to cirrhosis. Soluble mediators secreted from MSC suppress the proliferation and activation of various immune cells, which reduces the stimulation of pro-fibrosis factors secretion and alleviates fibrosis indirectly. MSC can secrete tumor necrosis factor alpha (TNF-a), interleukin (IL-10), transforming growth factor beta 3 (TGF-β3), and hepatocyte growth factor (HGF) to inhibit the proliferation of HSC and reduce ECM synthesis [10, 11]. Moreover, MSC-derived HGF can also accelerate the rate of HSC apoptosis. Hepatocytes differentiated from MSC will also contribute to improving liver function and histopathological grade. However, limited by the difficulty of locating the process, the transformation function requires further study.

To date, several clinical trials of MSC have been registered with ClinicalTrials.gov. The trials aim to evaluate the safety and efficacy of MSC to treat liver disease, such as liver cirrhosis and hepatic failure. Although trials with large patient cohorts and random controlled trials are not yet completed, the current data provide sufficient evidence for the potential of MSC to treat or ameliorate hepatic diseases.

The purpose of this study is to determine whether and how umbilical cord-derived MSC (UC-MSC) can improve the longer term survival of patients with liver cirrhosis. This study will also examine at how well UC-MSC are tolerated and their safety in patients with liver cirrhosis.

2 Objectives

Primary objective: To evaluate the efficacy of human UC-MSC to treat decompensated liver cirrhosis.

Secondary objective: To evaluate the safety of human UC-MSC in the treatment of decompensated liver cirrhosis

3 Study Design

3.1 Overall design and sample size

Overall design: This study follows the principle of Good Clinical Practice (GCP) to carry out a single center, open label, randomized controlled trial.

Participants: The participants are patients with decompensated liver cirrhosis associated with Chronic Hepatitis B (CHB). The total number of patients in this study is 250. After enrollment, the subjects are randomly assigned into the treatment group or the control group at a 1:1 ratio.

The administration regimens are as follows:

Treatment group: Conventional treatment + UC-MSC treatment, 0.5×10^6 UC-MSC/kg body weight, infused intravenously three times at 4-week intervals.

Control group: Conventional treatment according to the Chronic Hepatitis B Prevention and Treatment Guidelines (APASL 2008), the Cirrhotic Ascites Prevention and Treatment Guidelines (AASLD 2004), and the Cirrhotic Variceal Hemorrhage Prevention and Treatment Guidelines (AASLD 2008).

3.2 Endpoints

3.2.1 Primary endpoints

(1) Survival rate during 75 months posttreatment follow up.

(2) Hepatocellular carcinoma (HCC)-free survival rate during 75 months of posttreatment follow up.

3.2.2 Secondary endpoints

(1) Liver function, including the levels of albumin (ALB), prothrombin activity (PTA), total bilirubin (TBIL), and cholinesterase (CHE) during 48 weeks after the first UC-MSC infusion..

(2) Complications, including abdominal infection, gastrointestinal hemorrhage, hepatic encephalopathy, and hepatorenal syndrome during 48 weeks after the first UC-MSC infusion.

3.3 Randomization

Patients will be randomly allocated to the conventional treatment (control) or UC-MSC treatment (UC-MSC infusion) groups at a 1:1 ratio according to a computer-generated randomization list. The randomization list will be generated by third party statistical staff. The randomization code will be assigned to a participant at his or her first visit. The participants will be assigned to the treatment groups corresponding to the randomization code. This is an open-label study; both participants and the study team will be aware of the treatment allocation.

4 Participants

4.1 Inclusion Criteria

Subjects who meet all of the following conditions will be entered into the trial:

(1) Patients aged from 18 to 65 years.

(2) Testing positive for serum hepatitis B surface antigen (HBsAg) for more than 6 months.

(3) Diagnosis of decompensated liver cirrhosis, which was defined as liver cirrhosis with serious complications, such as ascites, hepatic encephalopathy, and esophageal and gastric variceal hemorrhage, according to Prevention and Treatment of Chronic Hepatitis B (China 2009).

(4) Negative pregnancy test (female patients of fertile age).

(5) Volunteering to participate in this clinical trial, and able to understand and provide signed informed consent.

(6) Patients with hepatitis B infection-related cirrhosis treated with anti-HBV therapy.

4.2 Exclusion Criteria

Subjects who meet one of the following conditions will not be allowed to enter the test:

(1) Hepatocellular carcinoma or other malignancies.

(2) Pregnancy or lactation.

(3) Sepsis.

(4) History of HCV infection.

(5) History of HIV infection.

(6) Other liver diseases, including, but not limited to, alcoholic liver disease, non-alcoholic fatty liver disease, drug-induced hepatitis, autoimmune hepatitis, Wilson disease, and hemochromatosis.

(7) ALT or AST $\geq$ 10*ULN.

(8) Creatinine clearance (CLcr) < 50 mL/min (calculated according to the Cockcroft-Gault formula) (Appendix 2).

(9) Diabetes mellitus (HbA1c > 8.5%).

(10) History or family history of mental or neural diseases, especially depression, depressive tendencies, epilepsy, and hysteria.

(11) Severe cardiovascular disease including, but not limited to, uncontrolled hypertension (systolic blood pressure ≥ 160 mmHg or diastolic blood pressure ≥ 100 mmHg), New York Heart Association (NYHA) cardiac function grade Ⅲ (Appendix 1) and above, unstable angina pectoris, myocardial infarction, or intracoronary angioplasty occurring within 6 months, QTc interval (QTcF or QTcB) ≥ 500 ms, II° or III° atrioventricular block, or other uncontrolled arrhythmias.

(12) Serious diseases of the blood system, including, but not limited to, various anemias and hemophilia.

(13) Serious digestive diseases including, but not limited to, gastrointestinal ulcers and colitis.

(14) Severe respiratory diseases including, but not limited to, pneumonia, pulmonary tuberculosis, chronic obstructive pulmonary disease, and interstitial pulmonary disease.

(15) Allergic constitution or history of severe allergies, especially allergies to stem cell products or ingredients.

(16) History of alcohol or drug abuse within 6 months.

(17) Participation in other clinical trials within 3 months.

(18) Unable or unwilling to provide informed consent or unable to comply with test requirements

(19) Other situations unsuitable to participation in the opinion of the researcher.

4.3 Withdrawal criteria

All participants who meet one of the following criteria would withdraw from the trial:

(1) Subject’s request to stop test for lack of efficacy, intolerance, adverse reactions, or withdrawal of informed consent by the participants.

(2) Poor compliance (unable to comply with the requirements of the test).

(3) Pregnancy or lactation for women during treatment.

(4) Any complication, adverse event (AE), or laboratory abnormality occurring during the

treatment, which in the opinion of the investigator, indicates that continued participation in the study is not in the best interest of the subject.

(5) Other conditions unsuitable for continuation of the test in the opinion of the investigator.

If participants withdraw from the study because of the occurrence of AEs or laboratory abnormalities, these important event and test results should be recorded in case report form (CRF).

The causes of shedding cases should be recorded in detail. Data involving safety and efficacy should be obtained as much as possible and all end-of-study assessments should be carried out with the subject's consent. All data should be kept intact for reference.

4.4 Termination criteria

The test shall be terminated if the following criteria are met:

(1) Serious safety problems occurred during the test.

(2) Termination requested by the unit responsible for clinical research (such as funding or management reasons).

(3) Termination requested by independent ethics committee or administrative department.

5 Interventions

5.1 Human mesenchymal stem cells

(1) Origin: UC-MSC derived from healthy fetal umbilical cords and processed by pulverization, centrifugation, culture, and activation *in vitro*.

(2) Culture: UC-MSC were prepared in an approved good manufacturing practice (GMP)-compliant facility and identified as described previously. In brief, with the written consent of the maternity patients, fresh human umbilical cords were obtained after birth and collected in cold Xeno-free MesenCult-XF medium (STEMCELL Technologies Inc., Vancouver, Canada). The mesenchymal tissues in the umbilical cord Wharton's jelly were diced into cubes of approximately 0.5 cm^3 and washed with Hanks' balanced saline solution (Gibco Invitrogen, Waltham, MA, USA). Then, the tissue pellets were seeded in a tissue culture flask (Corning Enterprises, Corning, NY, USA) in α-minimal essential medium (MEM) supplemented with 10% fetal bovine serum (STEMCELL Technologies). The medium was replenished every 3 days. The UC-MSC were cultured and collected between the third and fourth passages for infusion.

5.2 Conditions for preparation and monitoring

5.2.1 Umbilical cord collection

(1) Collection materials

① Collection bottle (2% gentamicin containing medium, 100 mL).

② Registration form.

③ Informed consent form.

④ Uniform marker.

⑤ Outer packing bag.

(2) Collection requirements

① Collection site

Collection of the umbilical cord should be carried out in the unit designated by the person in charge of the umbilical cord bank. The collection should be carried out strictly under aseptic conditions to prevent contamination with pathogens.

② Collection staff

The collection staff should go through strict education and training before they can collect the umbilical cords.

③ Collection materials

The materials in direct contact with the umbilical cord should comprise sterilized medical or scientific supplies. To prevent contamination with microorganisms, materials that are not in direct contact with the umbilical cord should also comprise disinfected supplies. In addition, to prevent contamination with foreign bodies other than pathogens, the use of materials with dust particles and short fibers should be avoided. Supplies for collection shall be carefully assembled by qualified staff in accordance with SOP and distributed to the collection site in advance.

(3) Collection method

① Collection time

The umbilical cord should be quickly collected after fetal delivery in an aseptic state.

② Collection before placental delivery

- Within 10 seconds after fetal delivery, clamp the umbilical cord at 5–8 cm near to the fetal navel using hemostatic forceps (to ensure that the fetal navel is intact), avoid contact with other objects as far as possible, and cut the umbilical cord outside of the hemostatic forceps.

- Put the broken end of the umbilical cord into a pre-prepared collection bottle, slide it slowly into the collection bottle, and then cut off the end connected to the placenta. Place the whole umbilical cord in the collection bottle and seal it.
- During collection after natural labor, the umbilical cord should be washed with 2% gentamicin-containing normal saline and loaded into the collection bottle to prevent secondary pollution.

③ **Collection after placental delivery**

- After fetal delivery, the umbilical cord was cut off from the fetal navel, and the fetus was taken away for normal treatment.
- Rinse umbilical cord with 2% gentamicin-containing normal saline to remove blood and meconium contamination.
- Put the umbilical cord in a pre-prepared collection bottle and seal it.

(4) Transportation requirements

① Transportation staff

The staff responsible for transporting the umbilical cord should be designated by the person in charge and have received strict education and training.

② Transportation conditions

- In the course of transportation, the ambient temperature should be 4–8 ℃, and the temperature change should not exceed 5 ℃.
- To meet the requirements of the transportation temperature, materials with thermal insulation function should be used for the container, and ice cubes should be loaded.

③ Transportation materials

- Transportation list.
- Tape.
- Thermal insulation box.

④ Transportation procedures

- Check the number of the umbilical cord.
- Check whether the collection bottle and related data are complete.
- Check the markers for accuracy.
- Fill in the number, date, and time on the transportation form, as well as the name of the transportation staff.
- Put ice bags and foam fillers in the thermal insulation box, to reach the required temperature.

- Avoid X-ray irradiation, temperature change, and excessive turbulence during transportation.
- The time from collection to separation shall not exceed 24 hours.

(5) Donator selection criteria

① Maternal requirements

- 36–42 weeks of pregnancy, normal development and nutrition.
- No infectious diseases (e.g., hepatitis B, hepatitis C, AIDS, or syphilis).
- No malignant tumors.
- No serious complications during pregnancy.
- No family history of hereditary diseases.

② Fetal requirements

- No malformations.
- No family history of hereditary diseases.

5.2.2 UC-MSC quality control

For quality control of the UC-MSC, the cultured cells at the third passage were examined for their phenotypes using flow cytometry analysis, for example, high expression of CD44, CD73, CD90, and CD105, but no expression of CD31, CD34, CD45, or HLA-DR. Alternatively, the digested cells were cultured in conditioned medium (STEMCELL Technologies), and subsequently cultured for osteogenesis and adipogenesis differentiation assays. Briefly, the digested cells were cultured in osteogenesis-inducing medium (Gibco Invitrogen) consisting of MesenCult basal medium, osteogenic stimulatory supplements, dexamethasone, β-glycerophosphate, and ascorbic acid ions for five weeks, or in adipogenic induction medium (Gibco Invitrogen) consisting of MesenCult basal medium and MesenCult adipogenic stimulatory supplements for two weeks. Subsequently, osteogenesis was assessed using alkaline phosphatase staining, and adipogenesis was assessed using oil red O staining. Moreover, the UC-MSC were confirmed as negative for all the tested contaminants before infusion, including *Mycoplasma* spp., gram-positive and gram-negative bacteria, and fungi. The endotoxin levels were below 5 EU/kg and viability was > 80%.

5.3 Used dosage of UC-MSC

The amount of UC-MSC was calculated according to 0.5×10^6 UC-MSC/kg body weight. UC-MSC were infused intravenously three times at 4-week intervals.

5.4 Treatment method and course of treatment

UC-MSC was suspended in 200 mL of saline and infused intravenously into the patient's forearm.

5.5 Conventional clinical treatment

Antiviral therapy in accordance with *the Guidelines for the prevention and treatment of chronic hepatitis B: APASL (2008 edition).*

The treatment of ascites, peritonitis, hepatorenal syndrome, and upper gastrointestinal bleeding was performed in accordance with the current domestic clinical routine treatment program.

6 Research process

All subjects are required to sign an informed consent form before screening. This study was divided into a screening period, a treatment period, and a follow-up period. The treatment period was 8 weeks and the follow-up period comprised about 73 months. Screening period: −7~0 days, treatment period: Week 0 (baseline), week 4, week 8; Follow-up periods: 2 weeks, 12 weeks, 24 weeks, 48 weeks, 24 months, 48 months, 60 months, and 75 months (see table 4). The specific visit items are as follows:

6．1 Follow-up process

Table 4 Follow-up flow chart

Visit item	Screening period	On treatment & Posttreatment visit period										
		Treatment 1	Visit 1	Treatment 2	Treatment 3	Visit 2	Visit 3	Visit 4	Visit 5	Visit 6	Visit 7	Visit 8
Visit time	−7~0 Day	Week 0	Week 2 ±2 days	Week 4 ±2 days	Week 8 ±7 days	Week 12 ±7 days	Week 24 ±14 days	Week 48 ±14 days	Month 24 ±15 days	Month 48 ±15 days	Month 60 ±30 days	Month 75 ±30 days
Informed consent	×											
Demographics	×											
Medical history	×											
Weight	×	×	×	×	×	×	×	×				
Vital signs	×	×	×	×	×	×	×	×				
Physical examination	×	×	×	×	×	×	×	×				
Urine pregnancy test (female)	×		×		×	×	×					
Routine blood test	×		×	×	×	×	×	×				

Visit item	Screening period	On treatment & Posttreatment visit period										
		Treatment 1	Visit 1	Treatment 2	Treatment 3	Visit 2	Visit 3	Visit 4	Visit 5	Visit 6	Visit 7	Visit 8
Visit time	−7~0 Day	Week 0	Week 2 ±2 days	Week 4 ±2 days	Week 8 ±7 days	Week 12 ±7 days	Week 24 ±14 days	Week 48 ±14 days	Month 24 ±15 days	Month 48 ±15 days	Month 60 ±30 days	Month 75 ±30 days
Routine urine test	×		×	×	×	×	×	×				
Biochemical test	×		×	×	×	×	×	×				
Coagulation function	×		×	×	×	×	×	×				
HbA1c	×											
Five items of hepatitis B	×											
HBV DNA	×					×	×	×				
AFP	×					×	×	×				
Anti-HCV	×											
Anti-HIV	×											
Abdominal ultrasound	×					×	×	×				

Visit item	Screening period	On treatment & Posttreatment visit period										
		Treatment 1	Visit 1	Treatment 2	Treatment 3	Visit 2	Visit 3	Visit 4	Visit 5	Visit 6	Visit 7	Visit 8
Visit time	−7~0 Day	Week 0	Week 2 ±2 days	Week 4 ±2 days	Week 8 ±7 days	Week 12 ±7 days	Week 24 ±14 days	Week 48 ±14 days	Month 24 ±15 days	Month 48 ±15 days	Month 60 ±30 days	Month 75 ±30 days
Chest X-ray	×											
Electrocardiogram (ECG)	×											
Adverse events (AEs)			×	×	×	×	×	×				
Complications			×	×	×	×	×	×				
UC-MSC infusion		×		×	×							
survival status (follow-up by telephone)									×	×	×	×
Occurrence HCC (follow-up by telephone)									×	×	×	×

6.2 Screening period: (−7~0 days)

(1) Signing of the informed consent form

The investigator or subinvestigator will fully explain the nature of the study to a subject using the institutional review board (IRB)-approved informed consent document. If the subject agrees to participate in the study, the subject must voluntarily sign an informed consent form before the initiation of any study procedures. A copy of the signed and dated informed consent document will be given to the subject. The signed and dated original consent form will be retained by the investigator. Informed consent must be obtained from all subjects. A subject cannot be entered into the study until he or she has signed and dated on the consent form. The investigator or subinvestigator is responsible for ensuring that the subject understands the risks and benefits of participating in the study and should answer any questions the subject may have during the discussion of informed consent performed before study initiation, as well as during the study period. The investigator or subinvestigator is also responsible for sharing any new information in a timely manner that might affect the subject's willingness to continue his or her participation in the study.

(2) Ask for demographic information and past medical history

The following baseline subject characteristics will be obtained and entered in the CRF: Date of written informed consent by the subject, date of birth, sex, ethnicity, race, inpatient or outpatient status, smoking habit, drinking habit, prior therapy, and medical history. Medical history will include any previous medical condition requiring hospitalization, all concurrent medical conditions, surgical history (within 12 months), time of influenza vaccination (within 12 months), diagnosis of influenza virus infection, and time of onset.

(3) Weight measurement

Weight measurement will be performed before treatment and during each visit, in accordance with the requirements of the study process. The weight test will be performed by the researchers.

(4) Physical examination and vital signs

Physical examination (see table 5) and vital signs (see tables 5 and 6) will be performed before treatment and during each visit, in accordance with the requirements of the study process. The

physical examination is completed by the researcher and judged by the researcher. Blood sample collection and processing will be performed in accordance with standard operating procedures (Appendix 3)

Table 5 Physical examination item list

**Physical examination**	**Indicators**	**Testing organization**
Vital signs	Body temperature (Tm), respiratory (Br), pulse (Pu), blood pressure (systolic blood pressure SBP, diastolic blood pressure DBP).	302 Hospital
Physical examination	Skin and mucosa, lymph nodes, facial features (head, eyes, ears, nose, pharynx), neck, chest, abdomen, spine, limbs, and others.	302 Hospital
Pregnancy examination. (women only)	Urinary pregnancy examination.	302 Hospital
Routine blood tests	Leukocyte (WBC), percentage of neutrophils (NE%), absolute value of neutrophils (NEU#), percentage of lymphocytes (LY%), absolute value of lymphocytes (LYM#), percentage of monocytes (MO%), absolute value of monocytes (MON#), percentage of eosinophils (EO%), absolute value of eosinophils (EO#), percentage of basophils (BA%), absolute value of basophils (BAS#), erythrocyte (RBC), hemoglobin (HGB), and Platelets (PLT)	302 Hospital
Routine urine tests	PH (PH), leukocyte (LEU), nitrite (NIT), protein (PRO), glucose (GLU), ketone body (KET), urinary bilirubin (UBG), bilirubin (BIL), erythrocyte (ERY), urinary sediment microscopic examination of leukocytes (WBC/HP), and microscopic examination of red blood cells (RBC/HP) with urinary sediment.	302 Hospital
Blood	Liver function: Total protein (TP), albumin (ALB), direct bilirubin	302 Hospital

biochemistry	(DBIL), total bilirubin (TBIL), alanine aminotransferase (ALT), aspartate aminotransferase (AST), cholinesterase (CHE).<br>Renal function: Urea (UREA), creatinine (CRE).<br>Blood glucose, blood lipid: Glucose (GLU), triglyceride (TG), and total cholesterol (TC).	
Coagulation function	Prothrombin time (PT), prothrombin activity (PTA), and international standardized ratio (INR)	302 Hospital
Upper abdominal ultrasound	Liver, gallbladder, spleen, pancreas, and kidney	302 Hospital
HbA1c	Glycosylated hemoglobin	302 Hospital
HBV tests	Serum: HBsAg/anti-HBs, HBeAg/anti-HBe, anti-HBcAg	302 Hospital
HBV DNA	Serum: HBV DNA	302 Hospital
Alpha fetoprotein	AFP	302 Hospital
anti-HCV	Serum: anti-HCV	302 Hospital
anti-HIV	Serum: anti-HIV	302 Hospital
Chest X-ray	Anterior and lateral chest position	302 Hospital
Electrocardiogram (ECG)	Heart rate (HR), P-R interval, QRS time, QT/ QTc, ECG diagnosis	302 Hospital
Adverse events	Evaluate according to the international NCI-CTC AE v4.0 standard	302 Hospital
Complications	Infection, gastrointestinal bleeding, encephalopathy, and hepatorenal syndrome	302 Hospital

Table 6. Normal range of vital signs

Vital signs	Normal range	
	Lower limit	Upper limit
Body temperature (armpit)	36 °C	37 °C
Breathing	16 times / min	20 times / min
Pulse	60 times / min	100 times / min
Blood pressure (diastolic blood pressure)	60 mmHg	90 mmHg
Blood pressure (systolic blood pressure)	90 mmHg	140 mmHg

(5) Pregnancy examination

In this study, women of childbearing age include: Female subjects younger than 50 years of age without menopause; Female subjects with menopause for ≤ 2 years and without surgical sterilization (bilateral tubal rolling, bilateral ovariectomy, or hysterectomy). Female partners who do not undergo surgical sterilization of male subjects are also considered women of childbearing age if they meet the above definition.

During the screening visit, women of childbearing age should be asked whether they have a history of menopause, what kind of contraception they use, and urine pregnancy tests should be carried out to determine whether they are qualified to join the group.

Female subjects of childbearing age should take effective contraceptive measures during the screening period, the treatment period, and within 16 weeks after the end of treatment (interview 5).

a. Time of collection of pregnancy information.

From the beginning of treatment (treatment 1) to 24 weeks (visit 3), information on all pregnancy events (including female partners of male subjects) at this stage should be collected. During each visit, the menstruation of female subjects of childbearing age should be enquired, such as delayed menstruation for 7 days or more, or delayed menstruation and did not take appropriate contraceptive measures (including unprotected sex); a urine pregnancy test should be performed.

Researchers may not actively collect pregnancy events within 16 to 40 weeks after the termination of MSC treatment (visits 3 to 4). If they know of any pregnancy events, they should inform the principal researchers in charge of the clinical study, including by email. Further treatment is carried out in accordance with the requirements of the Medical Ethics Committee and clinical trial institutions of the center.

b. Pregnancy report.

During treatment, once the female subject spontaneously reports or is later found that the female subject was pregnant or is likely to become pregnant, MSC should be discontinued immediately unless the researchers judged that the immediate cessation of MSC infusion would have serious medical consequences.

If any female subject or female partner of a male subject is found to be pregnant during the trial, the researcher should report to the unit responsible for the clinical study within 24 hours of being informed of her pregnancy and fill in the pregnancy report in a timely manner. The pregnancy of the subjects will be followed up until the end of the pregnancy (12 weeks after the due date) or the termination of the pregnancy, and the results will be reported to the unit responsible for the clinical study, which the researchers submitted as a follow-up report to the pregnancy report. Pregnancy events reported or found during the screening period should also be notified in writing (including e-mail) to the unit responsible for the clinical study and further dealt with in accordance with the requirements of the Medical Ethics Committee and the clinical trial institution of the center.

Pregnancy itself does not act as AE or SAE, but any complications during pregnancy (such as spontaneous abortion) or the choice of termination of pregnancy for medical reasons, and visits, will be recorded and followed up in accordance with the provisions of the program, and in accordance with AEs or SAEs. The pregnancy events of the female partners of the male subjects will be reported and followed up with reference to the pregnancy events of the female subjects.

(6) Routine blood tests

The routine blood tests will be completed by the clinical examination center of our hospital. The examination items are shown in Table 5. The results will be judged by the researchers.

(7) Routine urine tests

The urine routine tests will be completed by the clinical examination center of our hospital. The examination items are shown in Table 5. The results will be judged by the researchers.

(8) Coagulation function tests

The coagulation function tests will be completed by the clinical examination center of our hospital. The examination items are shown in Table 5. The results will be judged by the researchers.

(9) Glycosylated hemoglobin

The glycosylated hemoglobin test will be completed by the clinical examination center of our hospital. The results will be judged by the researchers.

(10) HBV tests

The HBV tests will be completed by the clinical examination center of our hospital. The results will be judged by the researchers.

(11) HBV DNA

The HBV DNA test will be completed by the clinical examination center of our hospital. The results will be judged by the researchers.

(12) AFP

The AFP test will be completed by the clinical examination center of our hospital. The results will be judged by the researchers.

(13) anti-HCV

The anti-HCV test will be completed by the clinical examination center of our hospital. The results will be judged by the researchers.

(14) anti-HIV

The anti-HIV test will be completed by the clinical examination center of our hospital. The results will be judged by the researchers.

(15) B-ultrasound examination of upper abdomen.

Professional doctors will provide diagnostic advice, and the results will be judged by the researchers.

(16) Chest X-ray

X-ray examination of anterior and lateral chest. Professional doctors will provide diagnostic advice, and the results will be judged by the researchers.

(17) Electrocardiographic examination

According to the requirements of the study process, a 12-lead ECG examination will be performed before treatment and during each visit. The ECG contents include the heart rate (HR), P-R interval, QRS time, QT/ QTc, ECG diagnosis (see Table 5). Professional doctors will provide diagnostic advice, and the results will be judged by the researchers.

6.3 Treatment 1 (week 0) baseline phase

(I) The verification of inclusion criteria and exclusion criteria should be carried out again;

(2) Weight measurement;

(3) Vital signs and physical examination;

(4) Treatment with UC-MSC.

6.4 Visit 1 (2nd week ±2 days)

(1) Weight measurement;

(2) Vital signs and physical examination;

(3) Urine pregnancy test for women of childbearing age with abnormal menstruation;

(4) Routine blood tests;

(5) Routine urine tests;

(6) Biochemical indexes: Liver function, renal function, blood glucose, and blood lipid;

(7) Coagulation function;

(8) Record AEs;

(9) Complications.

6.5 Treatment 2 (4th week ±2 days)

(1) Weight measurement;

(2) Vital signs and physical examination;

(3) Routine blood tests;

(4) Routine urine tests;

(5) Biochemical indexes: Liver function, renal function, blood glucose, and blood lipid;

(6) Coagulation function;

(7) Treatment with UC-MSC;

(8) Record AEs;

(9) Complications.

6.6 Treatment 3 (8th week ±7 days)

(1) Weight measurement;

(2) Vital signs and physical examination; urinary pregnancy test was performed in women of childbearing age, such as abnormal menstruation.

(3) Routine blood tests;

(4) Routine urine tests;

(5) Biochemical indexes: Liver function, renal function, blood glucose, and blood lipid;

(6) Coagulation function;

(7) Treatment with UC-MSC;

(8) Record AEs;

(9) Complications.

6.7 Visit 2 (12th week ±7 days)

(1) Weight measurement;

(2) Vital signs and physical examination;

(3) Urine pregnancy test for women of childbearing age with abnormal menstruation;

(4) Routine blood tests;

(5) Routine urine tests;

(6) Biochemical indexes: Liver function, renal function, blood glucose, and blood lipid;

(7) Coagulation function;

(8) HBV DNA quantification;

(9) AFP;

(10) Upper abdominal ultrasound;

(11) Record AEs;

(12) Complications.

6.8 Visit 3 (24th week ±14 days)

(1) Weight measurement;

(2) Vital signs and physical examination;

(3) Urine pregnancy test for women of childbearing age with abnormal menstruation;

(4) Routine blood tests;

(5) Routine urine tests;

(6) Biochemical indexes: Liver function, renal function, blood glucose, and blood lipid;

(7) Coagulation function;

(8) HBV DNA quantification;

(9) AFP;

(10) Upper abdominal ultrasound;

(11) Record AEs;

(12) Complications.

6.9 Visit 4 (48th week ±14 days)

(1) Weight measurement;

(2) Vital signs and physical examination;

(3) Routine blood tests;

(4) Routine urine tests;

(5) Biochemical indexes: Liver function, renal function, blood glucose, and blood lipid;

(6) Coagulation function;

(7) HBV DNA quantification;

(8) AFP;

(9) Upper abdominal ultrasound;

(10) Record AEs;

(11) Complications.

6.10 Visit 5 (24 months ±15 days)

(1) Survival status: Telephone call t;

(2) Whether liver cancer occurs or not: Telephone call t.

6.11 Visit 6 (48 months ±15 days)

(1) Survival status: Telephone call;

(2) Whether liver cancer occurs or not: Telephone call.

6.12 Visit 7 (60 months ±30 days)

(1) Survival status: Telephone visit;

(2) Whether liver cancer occurs or not: Telephone call t.

6.13 Visit 8 (75 months ± 30 days)

(1) Survival status: Telephone call;

(2) Whether liver cancer occurs or not: Telephone call.

6.14 Unplanned visits (when the study requires it or after withdrawal from the trial ahead of time)

If the subject needs additional visits (such as follow-up monitoring of adverse reactions or AEs) or withdraws from the trial ahead of time, the laboratory and auxiliary examinations should be improved as much as possible, recording AEs and the accompanying treatment. The measures taken by the researchers, including the results of laboratory tests, should be recorded in the original medical records and in the planned out-of-plan visit table in the CRF.

7 Efficacy Assessments

7.1 Survival analysis

The patients will be followed up for 75 months to record their survival status and survival rate after their first UC-MSC infusion. During the follow-up period, the survival rate of those patients is dependent on the survival status at the last visit point after the first infusion. The time of death of those patients will be recorded and the survival rate will be calculated.

7.2 Secondary Efficacy Assessments

Liver function (including the levels of albumin [ALB], prothrombin activity [PTA], total bilirubin [TBIL], and CHE) will be evaluated at 12, 24, and 48 weeks after the first UC-MSC

infusion.

The complications assessment will be followed-up for 48 weeks post-treatment (including infection, gastrointestinal bleeding, encephalopathy, and hepatorenal syndrome).

The diagnosis of complications

The diagnosis of ascites and hepatorenal syndrome will be made according to the AASLD ascites guidelines for liver cirrhosis (2004).

The diagnosis and treatment of hemorrhage and cirrhotic variceal bleeding will be made according to the AASLD guidelines for liver cirrhosis (2008).

The diagnosis of hepatic encephalopathy will be made according to guidelines of the 11th World Congress of Gastroenterology (WCOG), a working group that was established to discuss and summarize hepatic encephalopathy, and "The definition, nomenclature, diagnosis and quantitative analysis of hepatic encephalopathy" was published in the journal of the American society of hepatology in 2002.

8 Concomitant Diseases and Treatments

8.1 Concomitant diseases

If other diseases (excepting HBV infection and related diseases) are found in patients before signing informed consent, they would be considered as comorbid diseases and will be recorded on the CRF.

8.2 Concomitant treatments

Except for infusion of UC-MSC, all other treatments given to subjects at the time of entry into the trial or at any time during the trial will be considered concomitant treatment and the drugs used should be recorded in the CRF under their generic names. The concomitant drugs must be necessary for the subjects to use during the trial. The use of concomitant drugs could be allowed if the researchers consider that those drugs have no influence on UC-MSC efficacy. Meanwhile, the dose of the concomitant drugs should be kept to a minimum.

9 Safety evaluation

It is the responsibility of the researchers to identify and record events that meet the definitions of adverse events (AEs) and serious adverse events (SAEs) specified in this study.

Long-term safety is a major concern for this trial. Hepatocellular carcinoma (HCC) occurrence is defined as the long-term adverse events in this clinical trial. The duration between the first infusion and timepoint of HCC occurrence will be recorded during 75 months of follow-up. This duration will be defined as survival rate of patients without HCC.

9.1 Definition of AEs

An AE is defined as an untoward medical occurrence in a participant in this clinical trial that does not necessarily have a causal relationship with this treatment. Hence, any new unfavorable or unexpected sign (such as an abnormal laboratory finding), symptom, or disease temporally associated with the use of study drug, whether or not considered related to the study drug.

9.2 Definition of SAE

An SAE is any unexpected medical events occurrence that at any dose:

(1) Causes death;

(2) Is life-threatening (defined as an event in which the subject was at risk of death at the time of the event; it does not refer to an event which hypothetically might have caused death if it were more severe);

(3) Results in hospitalization or prolonged hospitalization.

In general, "hospitalization" means that the subject is not suitable for observation or treatment in the outpatient or emergency department, but requires formal admission or emergency observation (at least overnight). A complication that occurs during hospitalization is defined as an AE. If the length of stay in a participant is extended or any symptoms is met other than the SAE standard, it is also defined as SAE. If the patient needs elective surgery for a previous disease and the disease does not progress from the baseline, these conditions do not define an SAE;

(4) Disability, which refers to a person's substantial loss of normal living ability, excluding minor symptoms such as simple headache, nausea, vomiting, diarrhea, influenza, accidental injury, etc. Although these symptoms may have an impact on the ability of daily life, they do not

represent substantial or long-term living ability loss;

(5) Congenital malformations or Congenital anomaly.

Some medical or scientific judgment is needed to determine whether urgent reporting is required in such a situation. Medical event(s) that might not be immediately life-threatening or result in death or hospitalization but, based upon appropriate medical and scientific judgment, might jeopardize the subject or might require medical or surgical intervention to prevent one of the other serious outcomes listed in the definition above, which are defined as important medical events. Examples of such events include invasive or malignant cancer; allergic bronchospasm requiring intensive care in the emergency room or home; hematodyscrasia or convulsions before been hospitalized; and drug dependence or drug abuse.

In this study, the assessment of adverse events (AE) refers to the evaluation of the occurrence and severity of adverse events through medical history, vital signs, physical examination, and laboratory examination within 24 weeks after the first infusion of UC-MSC, which includes serious and important AE evaluation.

9.3 Abnormal clinical test or other abnormal indicators as adverse events or serious adverse events

If the researchers considered that abnormal laboratory tests (such as clinical biochemistry, hematology, urine analysis) were clinically significant, or other abnormal indicators (such as electrocardiogram, vital signs, or physical examination.) were clinically significant and met the definition of an AE or SAE, it would be recorded as an AE or SAE as well as being described by the symptoms and signs of the subjects. It should be regarded as AE or SAE if the clinically significant laboratory abnormalities and other abnormalities were found after the use of UC-MSC, or at the time of baseline assessment and are aggravated after the beginning of the study. However, the results of clinically significant abnormal laboratory tests or other abnormalities associated with the diseases would not be regarded as AE or SAE unless the researchers determined that the condition of the subject became more serious than expected or required medical treatment (such as withdrawal from this clinical trial or medical intervention). If laboratory findings or other anomalies that were present or found at the beginning of the study

are not aggravated, these events would be not included in AE and SAE.

Researchers will determine the clinical significance of an abnormal laboratory examination or other abnormalities according to the US national cancer institute standard and the actual situation of the subjects. All abnormal laboratory test results will be listed by the data statistician after the trail; meanwhile, the severity of those results is evaluated according to the NCI standard.

9.4 AE and SAE Collection and Recording

When an AE or SAE occurs, researchers have a responsibility to review all the relevant records (such as course records, laboratory tests, and diagnostic reports) and to record event-related data in the subjects' CRF.

Researchers should try their best to judge events based on symptoms, signs, or other clinical data. In this case, the diagnosis should be a specific AE or SAE and be recorded as such, not a single signs or symptoms of the subject.

9.5 AE and SAE assessment

9.5.1 Severity determination of an AE

Researchers should assess the severity of each reported AE and SAE based on their clinical knowledge and professional guidelines. The AE recorded in CRF should be classified according to the NCI-CTC AE 4.0 standard (see Appendix 4 for common AE grading standards):

Grade 1. Mild: Patients are asymptomatic or present mild signs; only clinical or diagnosis observation results are obtained; there is no need for interventional therapy;

Grade 2. Moderate: Patients need minimum, local, or non-invasive treatment; the ability of their instrumental daily life activities * is limited;

Grade 3. Serious or clinically significant, but not immediately life-threatening; hospitalization or prolonged hospitalization; disability; limited ability to take care of oneself via daily activities **

Grade 4: The abnormality leads to life-threatening consequences; the need for urgent intervention by clinical therapy;

Grade 5: An AE leads to death.

Illustration: The semicolon used in grade description mentioned above means “or”;

A single dash means that this Grade does not exist;

Not all AE contain all levels. Thus, some AEs have less than five Grades to choose from;

Grade 5 (death) does not apply to some AEs, so there is no such level;

Activities of daily living (ADL);

*Instrumental daily life activities include, for example, cooking, shopping, using the telephone, and money management.

** Personal daily activities include, for example, bathing, undressing, eating, washing, taking medicine, and the patients is not bedridden.

9.5.2 The judgment of causality

The researchers should judge the relationship between each AE and SAE and UC-MSC treatment based on their clinical experience. The relationship between an AE and UC-MSC treatment will be evaluated according to five grades: Surely related to, probably related to, might be related to, might not be related to, and surely not related to.

Adverse reactions refer to the sum of the first three grades. Other reasons, such as the natural history of potential diseases, other simultaneous treatments, risk factors, and temporary events related to UC-MSC treatment, need to be considered and examined. Researchers should also consult the handbook of clinical researchers and the UC-MSC treatment-related data for evaluation.

9.6 AE and SAE follow-up

Researchers should collect the records of AEs an SAEs at the beginning of signing informed consent to the completion of follow-up by spontaneous reports or medical examinations. Screening reports or findings of AEs and SAEs should also be reported in writing (including e-mail) by the unit responsible for clinical research and further processed in accordance with the requirements of the Medical Ethics Committee and clinical trial institutions of the center. Once the AE or SAE is reported, the researchers should interview each subject with the AE or SAE and provide the unit responsible for the clinical study with information about the event. All AEs and SAEs recorded that persisted from the previous visit or revisit should be reviewed during current

visit or revisit.

All patients with AEs and SAEs must be visited until their AE or SAE is alleviated, becomes stable, another explanation for the cause of the event is found, or until the patient is lost from follow-up. The AE and SAE part of the CRF should be updated timely when the problem is resolved. The researchers could add additional tests to the visit, which might help to clarify the nature or cause of the AE or SAE. This might include additional laboratory tests or studies, pathological examinations, or consultations with other professionals.

The unit in charge of the clinical research might require the researcher to complete or arrange additional examinations or assessments that could help to clarify the nature or causes of an AE or SAE, and the researcher is obliged to assist. If subjects die during participation in the study or an approved visit period, their autopsy results, including histopathological examination, should be provided to the unit responsible for the clinical study. New or updated data should be recorded on the initially completed CRF SAE page, and the researcher should sign and date the new or updated data. The CRF SAE update page shall be submitted to the unit responsible for clinical research in accordance with the provisions of 9.6.1.

9.6.1 SAE Reporting

As soon as the researchers realize that the subjects have developed an SAE, they have to undertake immediate treatment and report to the unit responsible for the clinical study within 24 hours. The SAE report should be record all available event-related information as completely as possible, be signed by the researcher (or designated person), and submitted to the unit responsible within a specified period of time. Even if the researchers did not obtain all the data on the SAE, they could report to the unit responsible for the clinical study on the relevant pages of the CRF and do not need to wait for other results. The CRF page of SAE can be updated when additional information is available.

In the initial report, the researchers should assess causality based on section 9.5.2 (judgment of causality). Researchers can send the SAE report by fax, which is the best way to submit this information to the person in charge of the research project. Telephone notification might be undertaken and a copy of the SAE report might be sent the next day in the absence of facsimile

equipment. However, telephone notification cannot replace the researchers' completed and signed SAE report.

9.6.2 AEs and SAEs after the study

AEs and SAEs after the study refers to any event that occurs outside the visit period of the AE or SAE specified in 9. 3 (method, frequency, and time of discovery of an AE or SAE). Researchers do not have to take the initiative to explore AE and SAE in subjects who had completed clinical studies and interviews. However, if the researcher is aware of any SAE (including death) at any time after a subject has withdrawn from the study, and the event might be related to the MSC, the researcher should promptly notify the unit responsible for the clinical study.

9.6.3 AE and SAE related to participation in the trial

SAE considered to be related to participation in the study (such as procedures, traumatic examinations, and changes in existing treatment options) should be reported promptly to the unit in charge of the clinical study, both before and after treatment.

10 Withdrawal from the study

10.1 Subjects accomplish the trail

We plan to enroll 250 patients in the trial. Participants will be deemed to have completed the clinical trial if they complete all treatments and visit periods (from the screening period to visit 8) as per the study protocol.

10.2 Subjects withdraw from the trial

Each participant may, at any time of the study, withdraw from the study for any reason (special or non-special) without being unfairly treated. The subjects who withdraw from the study will not be restricted in their treatment and may continue to receive conventional treatment or other appropriate treatment.

The medical examinations in their screening period should be cancelled for the enrolled patients who fail to be screened into the study, and the reasons for failure should be recorded in the completion/termination page.

If the successfully screened participants withdraw from the clinical trial during the study period, the medical examination for these subjects should be completed at the current visit, and the reasons for quitting the study should be recorded in the completion/termination page.

11 Data Management

This scheme only provides general requirements for data management. Data entry and modification: Data entry and management shall be performed by the data manager of the statistical unit, and will be managed by using an electronic database. To ensure the accuracy of the data, two data administrators are required to input and proofread the data independently.

For questions in the CRF, the data administrator should fill out the question-answer form and ask the researcher. The data administrator will modify, confirm, and enter the data according to the researcher's answers, and can issue the question-answer form again if necessary. The data administrator will import the data into the designated database, hand over the data to the statistician for statistical analysis, and the statistical analyst will write the statistical analysis report.

12 Statistical analysis

12.1 Sample size

The median disease-free survival was 60 months in 60% of the patients who received the standard medical treatment, and in 85% of those who received the UC-MSC therapy. Assuming a recruitment period of 40 months and a total follow-up period of over 75 months, with 100 patients in each group, the study would need to have power greater than 80% to detect a significant relative risk reduction, at a two-sided α level of 0.05 (PASS 11.0). Based on the dropout or withdrawal rate, we determined a sample size of 125 patients per group.

12.2 Definition and selection of analysis sets

This study used three data sets: A safety set, a full analysis set, and a per-protocol set.

Safety set (SS): SS refers to the set of all subjects who received at least one UC-MSC infusion and had a post-treatment safety record. The SS will be used for safety analysis and the subjects will be analyzed according to the actual treatment received.

Full analysis set (FAS): FAS refers to the set of all subjects who randomly received at least

one UC-MSC infusion. When the main efficacy indicators are missing, the missing data is not filled in, and will be assigned to the unresponsive set. FAS is the primary analysis set and will be used to report the distribution, demographic data, baseline characteristics, compliance, and efficacy evaluation for all subjects. The subjects will be analyzed in a randomized treatment group.

Per-protocol set (PPS): PPS refers to the set that the subjects coincide with the major inclusion criteria, do not coincide with the major exclusion criteria, complete the treatment regimen, do not take banned medication during the trial, and have no other major program violations. PPS will be used to evaluate primary efficacy.

12.3 Statistical methods

All statistical analysis will be performed using SAS 9.2 (SAS, Cary, NY, USA) or newer.

In general, continuous variables will be described statistically using the number of cases, mean, median, standard deviation, minimum, and maximum; normally distributed data will be expressed as the mean ± standard deviation, and skewed distribution data will be expressed as quartiles.

The categorical and grade variables will be described statistically using the frequency and percentage of each category or grade.

12.3.1 Case group analysis

(1) List the number of enrolled and completed clinical trial cases in each group will be listed, and the number and percentage of subjects in the three analytical data sets (SS, FAS, PPS) will be summarized;

(2) The number and proportion of subjects in each group who had early termination of trials for different reason will be summarized. The cases of shedding and removal, and their causes will be listed.

12.3.2 Demographic data and baseline analysis

Descriptive statistical demographic data and baseline characteristics:

(1) For the consecutive variables such as age, weight, vital signs, and the number of case, the mean, standard deviation, median, minimum, and maximum will be calculated.

(2) The count and grade data, such as gender, physical examination, will be used to calculate the frequency and composition ratio.

12.3.3 Safety Analysis

(1) AEs will be encoded according to MedDRA (International Medical Dictionary). The analysis of an AE will be based on the AE that occurs after UC-MSC treatment (TEAE). TEAE

is defined as an AE that occurs or worsens after UC-MSC treatment. For a TEAE in the treatment group, the TEAE may be related to UC-MSC treatment. An SAE will be analyzed according to the classification of system organs and preferred terms, and the number of occurrences and incidence rate will be calculated;

(2) Descriptive statistics will be used to summarize vital signs and laboratory tests. A shift table will be used to describe changes in normal/abnormal results in the laboratory tests before and after UC-MSC administration. In addition, the number and proportion of subjects will be calculated whose laboratory test are normal before treatment, but abnormal after treatment, or whose laboratory test are abnormal before treatment, but aggravated after treatment.

12.3.4 Efficacy analysis

The efficacy analysis will be based on the FAS set. PPS analysis will serve as an ancillary analysis for FAS. If the PPS results are inconsistent with the FAS results, it will be necessary to analyze and discuss the possible causes.

(1) Primary efficacy

All eligible participants who receive the assigned treatment and are followed up will be included in the analysis. We will count the number of endpoint events (death or HCC events) that occurred during the follow-up period and will compare the event-free time between the two groups using Kaplan–Meier analysis. The post-treatment follow-up will be continued until the date of an endpoint event, death, or the end of the study, whichever occurs first. Cox proportional-hazards models will be developed to estimate the hazard ratio (HR) and 95% confidence interval (CI) for between-group comparisons, with or without adjustments for stratification baseline MELD scores. Additionally, we will perform landmark analyses to assess the endpoint events accordingly, with the hazard ratio calculated separately for events that occurred before or after the landmark points.

(2) Secondary efficacy indicators

Liver function: The serum levels of ALB, PTA, CHE, and TBIL will be monitored within 48 weeks after the first UC-MSC infusion or routine medical treatment. A paired t-test will be applied to analyze the levels of ALB, PTA, CHE, and TBIL of the participants in each group post-treatment compared with those at baseline. The levels of ALB, PTA, CHE, and TBIL of the participants will also be compared between the treatment group and the control group at each specific evaluation timepoint. Student's t test or a non-parametric test will be applied when relevant.

Complications: MedDRA will be used to code and classify complications. For all cases, the

episode and severity of the complication will be listed by groups. The incidence by each study group and severity, respectively, will be calculated. The incidence between groups will be compared using the Chi-squared test or Fisher’s exact test. Multiple Chi-squared comparisons will be performed on the basis of a Bonferoni-adjusted α value when relevant.

12.4 Statistical software and general requirements

(1) Analysis will be performed using SAS 9.2 or newer;

(2) The estimated overall survival rate and HCC-free survival rate, as well as bilateral 95% confidence intervals, will be calculated;

(3) Unless there is another declaration, the hypothesis testing will be two-sided with an α value of 0.05.

13 Clinical trial management

13.1 Compliance with GCP requirements

This clinical trial will be carried out in accordance with the requirements of GCP in China.

13.2 Protecting the privacy of the subject

When filling out and managing the CRF, protecting the subject's privacy should be considered, for example, using a serial number to represent the subject. Moreover, the abbreviation of the subject's name can be used as a proxy for the name.

13.3 Informed consent form.

Before starting the study, the subject informed consent must be explained to each subject participating in the study by the investigator in a way that the subject can understand, and the subject's voluntary informed consent must be obtained in written form. The date of receipt of written informed consent should be entered into the CRF. The following information will be explained to the subjects:

(1) The purpose of the trial;

(2) The research process;

(3) Benefits and risks;

(4) Other alternative treatments;

(5) Subject rights: Subjects will not be adversely affected if they refuse to participate in this study;

(6) At any time, the subject may withdraw from the study without adverse effects;

(7) A confidentiality agreement;

(8) What the subject must follow;

(9) Anything that may protect the interests of the subject.

13.4 Program revision procedures

When it is necessary to modify the protocol or suspend the trial, the principal investigator should contact and consult with the unit responsible for clinical research. In the event of a decision to modify the protocol and suspend the trial, the principal investigator participating in the trial shall immediately inform all participants of the details and reasons. If a major revision is done, it should be implemented after approval by the Ethics Committee.

13.5 Study medical record and case report form record

Clinical trial data should be recorded truly and accurately.

Any modification in the medical records and CRFs must be clear and identifiable, and the date of the revision should be present. If the scope of the modification is large or the content of the modification is large, the modifier should indicate the date of modification and the reason for modification, and sign at the same time.

13.6 Quality control and quality assurance

(1) Clinical trial procedures shall follow standard operating procedures to ensure that the quality control system and quality assurance system for clinical trials are implemented.

(2) In all stages of clinical trials, a unified research protocol should be implemented.

(3) The same procedures are required to administer MSC in the trial at all stages of the trial, including receipt, storage, and distribution of MSC.

(4) All the observed results and abnormal findings in clinical trials should be carefully verified and recorded in a timely manner to ensure the reliability of the data. All kinds of instruments, equipment, reagents, and standard substances involved in the clinical trials must have strict quality standards, and ensure that they work under normal conditions. The recording and transfer of clinical data must be undertaken by experienced physicians and be supervised or checked by special personnel to ensure the science and accuracy of the data. The various conclusions of the clinical trial must be based on original data.

(5) The physician in charge of the experiment should fill in the study medical records and CRFs in a complete, detailed, accurate, and timely manner, and submit or keep them according to the prescribed procedures after being signed and confirmed by the superior physician. All clinical trial data should be centrally managed and analyzed.

(6) It is necessary to establish procedures for data storage, data transfer, and data query. Materials to be kept include: Study history, imaging data, CRF, MSC use registration form, subject screening form, SAE report form, GCP form to be completed by the hospital, interview

report form, and relevant original medical documents. The data to be delivered include: Subject CRF, SAE report form, and data needed for summary.

(7) Standard statistical analysis methods must be used to summarize and analyze the results of the clinical trial, and personnel familiar with biostatistics should be invited to participate.

13.7 Data storage

(1) The researcher must keep all research materials;

(2) The unit responsible and members of the clinical research team have the right to access or review all subjects' medical records after performing certain procedures;

(3) The retention period is 5 years after the end of the trial;

(4) The ownership of all data in this clinical trial belongs to the unit responsible for the clinical research. Except as required by the China Food and Drug Administration (cFDA), the researcher shall not provide the clinical trial data to any third party in any form without the written consent of the unit responsible for clinical research.

14 Risks and benefits

MSC are one of the most widely used stem cells in the world for clinical research because of their low immunogenicity, strong immunomodulation, good ability to repair tissue injury, wide sources, ease of access, and large-scale preparation. MSC have been applied to clinical studies of many diseases, showing good safety. Therefore, the safety risk and technical risk of MSC in the treatment of decompensated liver cirrhosis are low. Previous clinical studies have shown that MSC can improve the liver function and symptoms of patients with liver disease. This clinical study does not increase the financial burden of the patients, and will benefit patients with liver disease significantly.

15 Papers published

Participating clinical investigators shall keep confidential all data generated during the clinical study (except the medical records of the subjects) and shall not use the information, data, or records for purposes other than this clinical study.

These limitations do not apply if: (1) Publication of the data does not result from an error made by any of the participants; (2) Information that must be made public by an academic or ethics committee for the purpose of evaluating the study; (3) Information must be disclosed to

provide subjects with appropriate medical care.

The summary, submission, and publication of clinical research results shall be reviewed by the main researchers of the sponsor unit and the unit responsible for the clinical research, and shall be approved in writing.

16 Ethics

This study complies with the requirements of the Drug Clinical Trial Management Standard, and is strictly implemented in accordance with the requirements stipulated by the GCP of the cFDA. Before the implementation of the protocol, the subject shall be informed of the phase of the study, its possible effectiveness and possible risks, to ensure that the subject might withdraw from the clinical trial at any stage of the study for any reason without being unfairly treated, and the right to receive other treatments once the study is discontinued. The privacy of the subject will be strictly protected. The treatment with UC-MSC will be initiated after the subjects and their families have fully understood and signed on a voluntary basis; the rights and safety of the subject are protected at the same time. The umbilical cord has abundant resources and convenient materials completes its function immediately after the fetus is delivered and is normally "discarded", which will not cause any potential harm to the donor and will not involve ethical and moral issues for this research, which fully conforms to the ethical requirements.

17 References

1. Schuppan D, Afdhal NH. Liver cirrhosis. Lancet, 2008; 371(9615): 838-51.
2. Gronthos S, Zannettino AC, Hay SJ, Shi S, Graves SE, Kortesidis A, et al. Molecular and cellular characterisation of highly purified stromal stem cells derived from human bone marrow. J Cell Sci, 2003; 116(Pt 9):1827-35.
3. Lee OK, Kuo TK, Chen WM, Lee KD, Hsieh SL, Chen TH. Isolation of multipotent mesenchymal stem cells from umbilical cord blood. Blood, 2004; 103(5):1669-75.
4. Zuk PA, Zhu M, Mizuno H, Huang J, Futrell JW, Katz AJ, et al. Multilineage cells from human adipose tissue: implications for cell-based therapies. Tissue engineering, 2001; 7(2): 211-28.
5. Fukuchi Y, Nakajima H, Sugiyama D, Hirose I, Kitamura T, Tsuji K. Human placenta-derived

cells have mesenchymal stem/progenitor cell potential. Stem cells, 2004; 22(5): 649-58.

6. Dominici M, Le Blanc K, Mueller I, Slaper-Cortenbach I, Marini F, Krause D, et al. Minimal criteria for defining multipotent mesenchymal stromal cells. The International Society for Cellular Therapy position statement. Cytotherapy, 2006; 8(4): 315-7.
7. Kisseleva T, Gigante E, Brenner DA. Recent advances in liver stem cell therapy. Curr Opin Gastroenterol, 2010; 26(4): 395-402.
8. Kharaziha P, Hellström PM, Noorinayer B, Farzaneh F, Aghajani K, Jafari F, et al. Improvement of liver function in liver cirrhosis patients after autologous mesenchymal stem cell injection: a phase I-II clinical trial. Eur J Gastroenterol Hepatol, 2009; 21(10): 1199-205.
9. Mohamadnejad M, Alimoghaddam K, Mohyeddin-Bonab M, Bagheri M, Bashtar M, Ghanaati H, et al. Phase 1 trial of autologous bone marrow mesenchymal stem cell transplantation in patients with decompensated liver cirrhosis. Arch Iran Med, 2007; 10(4): 459-66.
10. Parekkadan B, van Poll D, Megeed Z, Kobayashi N, Tilles AW, Berthiaume F, et al. Immunomodulation of activated hepatic stellate cells by mesenchymal stem cells. Biochem Biophy Res Commu, 2007; 363(2): 247-52.
11. Wang J, Bian C, Liao L, Zhu Y, Li J, Zeng L, et al. Inhibition of hepatic stellate cells proliferation by mesenchymal stem cells and the possible mechanisms. Hepatology research: the official journal of the Japan Society of Hepatology, 2009; 39(12): 1219-28.
