# Supplementary material for: Mesenchymal stem cell therapy in decompensated liver cirrhosis: a long-term follow-up analysis of the randomized controlled clinical trial
Source: Hepatol Int. 2021 Nov 29;15(6):1431–41. doi: 10.1007/s12072-021-10199-2 (PMC8651584; doi:10.1007/s12072-021-10199-2)
Supplement: Supplementary file 2 — Supplementary file2 (PDF 510 kb) [file 12072_2021_10199_MOESM2_ESM.pdf]

Appendix 1

New York Heart Association (NYHA) Classification Standards

Class I: The patient has a heart attack but their activity is not restricted. Ordinary physical activity does not cause fatigue, palpitations, dyspnea, or angina.

Class II (I degree of heart failure): The physical activity of patients with heart disease is mildly restricted and there are no conscious symptoms at rest; however, fatigue, palpitations, dyspnea, or angina pectoris might occur under normal activities.

Class III (II degree of heart failure): The physical activity of patients with heart disease is obviously limited, to a degree that is less than the normal activities that usually cause the above symptoms.

Class IV (III degree of heart failure): The patients with heart disease cannot engage in any physical activity. Symptoms of heart failure also occur during rest, which worsen after physical activity.

Appendix 2

Cockcroft-Gault formula for the creatinine clearance rate (CLcr)

When the unit of serum creatinine is mg/dl, the calculation formula is:

CLcr (ml/min) = (male) (140−age) × weight (kg) / [72 × serum creatinine] (mg/dl)

(Female) (140−age) × weight (kg) / [72 × serum creatinine] (mg/dl) ×0.85

Conversion for different units of serum creatinine: mg/dl × 88.4 = μmol/l

μmol/l × 0.01131 = mg/dl

Appendix 3

Standard operating procedures for blood sample collection and processing

1. Purpose: To describe standard operating procedures for the collection and processing of test blood samples.
2. Scope: The collection and processing of blood samples from all subjects in this project, and the clinical site of the center is in charge of the specific work.
3. Operating procedures

3.1 Collection of blood samples

3.1.1 Equipment and consumables

Latex gloves, work clothes, hat, butterfly blood collection needle, tourniquet, sterile vacuum blood collection tube (coagulant, EDTA anticoagulation), blood collection sleeve, sterile gauze, medical alcohol cotton ball, sterile cotton ball, iodophor, blood collection pillow, disposable paper pad, and test tube rack.

3.1.2 Blood collection method

(1) Blood collectors wear work clothes, wash their hands, and wear glove, and hats;

(2) Clean the blood collection work surface with 70% alcohol, and put a disposable paper pad on the blood collection pillow;

(3) Check the identity of the blood sampled;

(4) Prepare blood collection tubes of appropriate specifications;

(5) Let the patient sit on the stool, exposing one arm to the upper arm; the cuffs should not be too tight;

(6) Have the patient place their arms on the blood-collection pillow with their palms facing upwards and fasten their upper arms with a tourniquet. The elbows should be below the heart level line;

(7) Look for a bulging vein in the anterior elbow area, and then check that the vein is well fixed to the surrounding tissue, touch and confirm the vein;

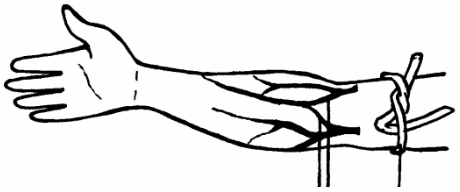

Median cubital vein   Tourniquet

(8) Disinfect the collection site in concentric circles with iodophors, and do not touch the disinfected parts with your hands;

(9) Rotate the screw port on the side of the blood collection needle with the blood collection sleeve and tighten;

(10) Ask the patient to make a strong fist; at the position where the blood is taken into the needle, fix the vein with the left hand;

(11) Insert the needle into the vein at 15° to the forearm along the blood flow direction; push the blood collection tube in the direction of the blood collection cannula;

(12) As the blood automatically flows into the blood collection tube, ask the patient to slowly releases his/her fist;

(13) After the blood collection tube is filled, the blood flow is automatically stopped, and another vacuum blood collection tube is withdrawn and replaced;

(14) Gently shake the blood collection tube after blood collection has been completed, so that the collected blood is in full contact with the anticoagulant and anticoagulation;

(15) After the blood collection is completed, loosen the tourniquet and quickly pull out the blood collection needle, while holding the blood collection wound with a sterile cotton ball;

(16) Ask the patient to bend their elbow and hold the cotton ball for more than 5 minutes, with the arm raised above the heart level line;

(17) Insert the blood collection tubes upright into the test tube rack in order;

(18) Discard the needle into a sharps container, and place disposable paper pad, etc. into a clinical waste container;

(19) Fill in the sampling record form, and sign and date it.

Note: If there is a hematoma, loosen the tourniquet and pull out the blood collection needle. After the first blood collection fails, another blood collection is allowed.

3.2 Handling and storing of blood samples: Please refer to the service manual of the central laboratory for details.

Appendix 4

Toxicity classification of adverse events corresponding to the screening items selected according to this study (National Cancer Institute Common Terminology Criteria Adverse Events, NCI-CTC AE v4.0)

Various inspections					
	(Grade)				
**AE**	**1**	**2**	**3**	**4**	**5**
Leukopenia	< the lower limit of the normal value to 3000/mm^3; < lower limit of the normal value to 3.0 × 10e9/l	< 3000–2000/mm^3; < 3.0– 2.0 × 10e9/l	< 2000–1000/mm^3; < 2.0–1.0 × 10e9/l	< 1000/mm^3; < 1.0 × 10e9/l	—
Definition: Laboratory tests have shown that the number of white blood cells in blood samples is reduced.					
Reduced platelet count	< the lower limit of the normal value to 75,000/mm^3; < lower limit of the normal value to 75.0 × 10e9/l	< 75,000– 50,000/mm^3; < 75.0–50.0 × 10e9/l	< 50,000– 25,000/mm^3; < 50.0–25.0 × 10e9 /l	< 25,000/mm^3; < 25.0 × 10e9/l	—
Definition: Clinical tests have shown that platelet counts in the blood are reduced.					
Reduced neutrophil count	< the lower limit of the normal value to 1500/mm^3; < the lower limit of the normal value to 1.5 × 10e9/l	< 1500–1000/mm^3; < 1.5–1.0 × 10e9 /l	< 1000– 500/mm^3; < 1.0–0.5 × 10e9 /l	< 500/mm^3; < 0.5 × 10e9/l	—
Definition: Clinical tests have shown that the number of neutrophils in the blood is reduced.					

Various inspections					
	(Grade)				
**AE**	**1**	**2**	**3**	**4**	**5**
Anemia	Hemoglobin < the lower limit of the normal value to 10.0 g/dl; < the lower limit of the normal value to 6.2 mmol/l; < the lower limit of the normal value to 100 g/l	Hemoglobin <10.0–8.0 g/dl; < 6.2–4.9 mmol/l; <100–80 g/l	Hemoglobin < 8.0–6.5 g/dl; < 4.9–4.0 mmol/l; <80–65 g/l; Need blood transfusion treatment	Life-threatening; urgent treatment needed	Death
Definition: Diseases characterized by a decrease in total hemoglobin in 100 ml of blood. Signs and symptoms of anemia include: Pale skin and mucous membranes, shortness of breath, palpitations, soft systolic murmurs, lethargy, and fatigue.					
Alanine aminotransferase increased	> The upper limit of normal value to 3.0 times the upper normal value	Asymptomatic: > 3.0–5.0 times the upper limit of the normal value; > 3.0 times the upper limit of the normal value, accompanied by worsening of the following symptoms: Fatigue, nausea, vomiting, pain or tenderness in the upper right area, fever, rash, and eosinophilia	> 5.0–20.0 times the upper limit of the normal value; for more than 2 weeks, > 5 times the upper limit of the normal value	> 20.0 times the upper limit of the normal value	—
Definition: Laboratory tests have shown increased alanine transferase (ALT) levels in blood samples.					

Various inspections					
	(Grade)				
**AE**	**1**	**2**	**3**	**4**	**5**
Aspartate aminotransferase increased	> The upper limit of the normal value to 3.0 times the upper normal value	Asymptomatic: > 3.0–5.0 times the upper limit of the normal value; > 3 times the upper limit of the normal value, accompanied by worsening of the following symptoms: Fatigue, nausea, vomiting, pain or tenderness in the upper right area, fever, rash, and eosinophilia	> 5.0–20.0 times the upper limit of the normal value; for more than 2 weeks, > 5 times the upper limit of the normal value	> 20.0 times the upper limit of the normal value	—
Definition: Laboratory tests have shown increased aspartate transferase (AST) levels in blood samples.					
Glutamyl transferase (GGT) increased	> The upper limit of the normal value to 2.5 times the upper normal value	> 2.5–5.0 times the upper normal value	> 5.0– 20.0 times the upper normal value	> 20.0 times the upper normal value	—
Definition: Laboratory tests have shown increased glutamyltransferase (GGT) levels in blood samples, exceeding normal levels. GGT catalyzes the transfer of the gamma amino group of a gamma glutamate polypeptide to another polypeptide, amino acid, or water.					
Alkaline phosphatase increased	> The upper limit of normal value to 2.5 times the upper normal value	> 2.5–5.0 times the upper normal value	> 5.0–20.0 times the upper normal value	> 20.0 times the upper normal value	—
Definition: Laboratory tests have shown increased alkaline phosphatase levels in blood samples.					

Various inspections

	(Grade)				
AE	1	2	3	4	5
Blood bilirubin increased	> The upper limit of the normal value to1.5 times the upper normal value	> 1.5 - 3.0 times the upper limit of the normal value	> 3.0–10.0 times the upper limit of the normal value	> 10.0 times the upper limit of the normal value	—
Definition: Laboratory tests have shown abnormally elevated bilirubin levels in blood samples and jaundice associated with excessive bilirubin.					
Hypoalbuminemia	< the lower limit of the normal value to 3 g/dl; < the lower limit of the normal value to 30 g/l;	< 3–2 g/dl; < 30–20 g/l	< 2 g/dl; < 20 g/l	Life-threatening; need treatment	Death
Definition: Laboratory tests have shown that the serum protein concentration in blood is lower than the normal value.					
Creatinine increased	> 1–1.5 times the baseline value; > the upper normal value to 1.5 × the upper limit of the normal value	> 1.5–3.0 times the baseline value; > 1.5–3.0 times the upper limit of the normal value	> 3.0 times the baseline value; > 3.0–6.0 times the upper limit of the normal value	> 6.0 times the upper limit of the normal value	—
Definition: Laboratory tests of biological samples show increased creatinine levels.					
Hyperglycemia	Fasting glucose concentration > the upper limit of the normal value-160 mg/dl; fasting glucose concentration > the upper limit of the normal value to 8.9 mmol/l	Fasting glucose concentration > 160–250 mg/dl; fasting glucose concentration > 8.9–13.9 mmol/l	> 250–500 mg/dl; > 13.9 –27.8 mmol/l; hospitalization required	> 500 mg/dl; > 27.8 mmol/l; Life-threatening	Death
Definition: Laboratory tests have shown that blood glucose levels are higher than normal and are usually an indicator of diabetes or glucose intolerance.					
Hypoglycemia	< the lower limit of the normal value to 55 mg/dl; < the lower limit of the normal value to 3.0	< 55–- 40 mg/dl; < 3.0–2.2 mmol/l	< 40– 30 mg/dl; < 2.2–1.7 mmol/l	< 30 mg/dl; < 1.7 mmol/l; life-threatening; seizures	Death

	mmol/l				
Definition: Laboratory tests have shown that the blood glucose concentration is below the normal value.					

**Various inspections**					
	**(Grade)**				
**AE**	**1**	**2**	**3**	**4**	**5**
Hypercholesterolemia	> the upper limit of the normal value to 300 mg/dl; > the upper limit of the normal value to 7.75 mmol/l	> 300–400 mg/dl; > 7.75–10.34 mmol/l	> 400– 500 g/dl; > 10.34 –12.92 mmol/l	> 500 mg/dl; > 12.92 mmol/l	—
Definition: Laboratory tests show that blood cholesterol levels are higher than the normal value.					
Hypertriglyceridemia	150 mg/dl–300 mg/dl; 1.71 mmol/l–3.42 mmol/l	> 300 mg/dl–500 mg/dl; > 3.42 mmol/l–5.7 mmol/l	> 500 mg/dl–1000 mg/dl; > 5.7 mmol/l–11.4 mmol/l	> 1000 mg/dl; > 11.4 mmol/l; life-threatening	Death
Definition: Laboratory tests have shown that the triglyceride concentration in the blood is higher than normal value.					
Hypokalemia	< the lower limit of the normal value to 3.0 mmol/l	< the lower limit of the normal value to 3.0 mmol/l; Asymptomatic: need treatment Symptomatic; need treatment	< 3.0–2.5 mmol/l; hospitalization required	< 2.5 mmol/l; life-threatening	Death
Definition: Laboratory tests have shown that the potassium concentration in the blood is below the normal value.					
Hyperkalemia	>the upper limit of the normal value =to 5.5 mmol/l	> 5.5–6.0 mmol/l	> 6.0–7.0 mmol/l; hospitalization required	> 7.0 mmol/l; life-threatening	Death
Definition: Laboratory tests have shown that the concentrations of potassium in the blood are higher than the normal value; elevated in kidney failure or sometimes diuretic use.					
Hyponatremia	< the lower limit of the normal value to 130 mmol/l	—	< 130–120 mmol/l	<120 mmol/l; life-threatening	Death

Definition: Laboratory tests have shown that the sodium concentration in the blood is below the normal value.

Various inspections					
	(Grade)				
AE	1	2	3	4	5
Hypernatremia	> the upper limit of the normal value to 150 mmol/l	150–55 mmol/l	> 155–160 mmol/l; hospitalization required	> 160 mmol/l; life-threatening	Death
Definition: Laboratory tests have shown that the sodium concentration in the blood is higher than normal value.					
Hypocalcemia	< the lower limit of the normal value- to 8.0 mg/dl; < the lower limit of the normal value to 2.0 mmol/l; calcium ion concentration < the lower limit of the normal value -1.0 mmol/l	< 8.0–7.0 mg/dl; < 2.0–1.75 mmol/l; calcium ion concentration < 1.0–0.9 mmol/l; Symptomatic	< 7.0–6.0 mg/dl; < 1.75 –1.5 mmol/l; calcium ion concentration < 0.9–0.8 mmol/l; hospitalization required	< 6.0 mg/dl; < 1.5 mmol/l; calcium ion concentration < 0.8 mmol/l; life-threatening	Death
Definition: Laboratory tests have shown that the concentration of calcium in the blood is below normal value.					
Hypercalcemia	> the upper limit of the normal value to 11.5 mg/dl; > the upper limit of the normal value to 2.9 mmol/l; Ionic calcium concentration >the upper limit of the normal value to 1.5 mmol/l	> 11.5–12.5 mg/dl; > 2.9 - 3.1mmol/l; Ionic calcium concentration >1.5–1.6 mmol/l; Symptomatic	> 12.5–13.5 mg/dl; > 3.1 –3.4 mmol/l; Ionic calcium concentration >1.6–1.8 mmol/l; hospitalization required	> 13.5 mg/dl; > 3.4 mmol/l; Ionic calcium concentration < 0.8 mmol/l; life-threatening	Death
Definition: Laboratory tests have shown that the calcium concentration in the blood is higher than the normal value.					
Proteinuria	Proteinuria 1+, 24-hour urine protein is less than 1.0 g	Adults: proteinuria 2+, 24-hour urine protein 1.0–3.4 g, children: Urine	Adult: 24-hour urine protein greater than 3.5 g, Child: Urine protein /	—	—

		protein / creatinine ratio 0.5-1.9	creatinine ratio greater than 1.9		
Definition: The laboratory tests find that too much protein is present in the urine, mainly albumin, but also globulin.					

**Various inspections**					
	**(Grade)**				
**AE**	**1**	**2**	**3**	**4**	**5**
Hematuria	Asymptomatic, only clinical observation or diagnosis, no treatment required	Minor symptoms requiring urinary catheter or bladder washing; affecting instrumental activities of daily living	Massive hematuria, requiring blood transfusion, intravenous medication or hospitalization; requiring selective endoscopic, radiotherapy or surgery; affects personal daily activities	Life-threatening; urgent radiology or surgery required	Death
Definition: Laboratory tests find blood in the urine.					
Investigations - Other, specify	Asymptomatic or mild; only clinical examination or diagnostic findings; no treatment required	Moderate symptoms; mild, local or non-invasive treatment; affects age-related instrumental activities of daily living	Severe or medical symptoms are obvious, but not immediately life-threatening; hospitalization or length of hospital	Life-threatening; urgent treatment needed	Death

			stay is required; disability; affects personal daily activities		

**Symptoms and Signs**					
	**(Grade)**				
**AE**	**1**	**2**	**3**	**4**	**5**
Fatigue	Relief after fatigue break	Fatigue, not relieved after rest; affects instrumental ADL	Affect individual ADL	—	—
Definition: The whole body is in a state of weakness, and it is not easy to arouse the spirit to complete the daily work.					
Headache	Mild headache	Moderate headache; affects instrumental ADL	Severe headache; affects individual ADL	—	—
Definition: The obvious discomfort in different areas of the head is not limited to the nerve distribution area.					
Nausea	Decreased appetite without changing eating habits	Reduced oral intake without significant weight loss, dehydration or malnutrition	Insufficient oral energy and water intake; requires nasal feeding, total parenteral nutrition or hospitalization	—	—
Definition: A condition characterized by nausea and / or vomiting.					
Insomnia	Mild sleep difficulty, keep sleeping or wake up early	Moderate sleep difficulty, keep sleeping or wake up early	Severe sleep difficulty, keep sleeping or wake up early	—	—
Definition: It is difficult to fall asleep or stay asleep.					
Anorexia	Loss of appetite, but	Changes in eating	Significant weight	Life-threatening;	Death

	without changes in eating habits	without accompanying weight loss or malnutrition; oral nutritional supplements required	loss or malnutrition (e.g. insufficient calorific intake) requiring nasal feeding or total parenteral nutrition	urgent treatment needed	
Definition: Loss of appetite.					

**Symptoms and Signs**					
**(Grade)**					
**AE**	**1**	**2**	**3**	**4**	**5**
Fever	38.0–39.0 ℃	> 39.0–40.0 ℃	> 40.0 ℃, ≤ 24 hours	> 40.0 ℃, >24 hours	Death
Definition: Body temperature is above the upper limit of normal value.					
Shiver	Slight cold sensation; tremor; tooth tremor	Moderate general tremor; requires narcotics	Severe or delayed or unresponsive to narcotics	—	—
Definition: A state of physiological reactions, usually a cold response after fever and sweating					
Diarrhea	Fecal frequency increased < 4 times per day compared with baseline; slight increase in fistula discharge	Fecal frequency increased to 4–6 times per day compared with baseline; moderate increase in fistula discharge	Fecal frequency increased ≥ 7 times per day compared with baseline; stool incontinence; hospitalization was required; severe increase in fistula discharge compared to baseline; affected individual ADL	Life-threatening; urgent treatment needed	Death
Definition: Frequent watery stool excretion.					
Myalgia	Mild pain	Moderate pain, affecting instrumental ADL	Acute pain, affecting individual ADL	—	—

Definition: Significant discomfort in one or a group of muscles					
**Symptoms and Signs**					
	**(Grade)**				
**AE**	**1**	**2**	**3**	**4**	**5**
Weight gain	With reference to the baseline, weight gain of 5 to < 10%	With reference to the baseline, weight gain of 10 to < 20%	With reference to the baseline, weight gain of ≥ 20%	—	—
Definition: For children, it means weight exceeds the growth curve baseline.					
Weight loss	With reference to the baseline, weight loss of 5 to < 10%, no treatment needed	With reference to the baseline, weight loss of 10 to < 20%, need for nutritional support	With reference to the baseline, weight loss of ≥ 20%, need nasal feeding or total parenteral nutrition	—	—
Definition: Weight loss: For children, it means weight is below the baseline value of the growth curve.					
Pruritus	Mild or limited: local treatment is required	Concentrated or widely distributed: intermittent attack; skin changes caused by scratching (swelling, papule, desquamation, moss like, exudation); need to be treated with oral medicine; influence on instrumental activities of daily living	Persistent attack; affect individual activities of daily living or sleep; need to take orally cortisone or immunosuppressant treatment	—	—
Definition: A condition characterized by intense itching.					
Acne-like rash	Pimples and pustules less than 10% of body surface area, with or without itching and tenderness	Pimples and pustules less than 10% of body surface area, with or without itching and tenderness; Associated	Pimples and pustules less than 10% of body surface area, with or without itching and tenderness; Affect	Pimples and pustules spread throughout the body surface and require intravenous antibiotics to treat a	Death

		with psychological effects; influences instrumental activities of daily living	individual activities of daily living; need oral antibiotics to treat double infection	wide range of multiple infections; life-threatening	
Definition: A condition characterized by pimples and pustules, mainly on the face, scalp, upper chest, and back.					
**Other**					
**(Grade)**					
**AE**	**1**	**2**	**3**	**4**	**5**
Maculopapule	Maculopapular rash covers less than 10% of the body surface area, with or without symptoms (e.g., itching, burning, tightness)	Maculopapular rash covers less than 10% of the body surface area, with or without symptoms (e.g., itching, burning, tightness); Affect instrumental ADL	Maculopapular rash covers less than 10% of the body surface area, with or without symptoms; Affect individual ADL	—	—
Definition: Macular rash and pimples appear. The common manifestations of skin damage often affect the upper body and develop centripetally with itching.					
Skin and subcutaneous diseases - Other, specify	Asymptomatic, mild symptoms, only diagnosed, no intervention required	Moderate symptoms; local / non-invasive intervention; affect age-adaptive instrumental ADL	Severe or medically significant but not critical symptoms; requiring hospitalization or prolonged hospitalization; affect individual ADL	Life-threatening; urgent treatment needed	Death
Flu like symptoms	Mild	Moderate; affect instrumental ADL	Severe symptoms, affect individual ADL	—	—
Definition: A series of illnesses with clinical symptoms similar to those of flu patients, including symptoms of fever, chills, general soreness, general weakness, anorexia, and dry mouth.					
Fidgety	Mild symptoms	Moderate; affect	Severe symptoms,	—	—

		instrumental ADL	affect individual ADL		
Definition: Cannot rest and relax.					

Clinical trial program confirmation signature page

Clinical research program of human mesenchymal stem cells in the treatment of decompensated liver cirrhosis

Consent from the principal investigator of the program:

I have read this program carefully. I agree with the inclusion of all the necessary information in the program to conduct the research, and I agree with the content described in the program. I understand that without the approval of the ethics committee, the trial must not be started, and the relevant regulations of the unit must be fully complied with.

The informed consent and corresponding record documents of all subjects participating in the trial are required. After signing the informed consent, clinical trials will be carried out according to the Helsinki Declaration and the requirements of laws and regulations on the clinical application of new drugs and approved drugs.

Name of principal investigator: ______________________

Name of research center: ____

____

Address of research center: ____

____

Signature of principal investigator: ________________ Date: ____________________

Clinical trial program confirmation signature page

Clinical research program of human mesenchymal stem cells in the treatment of decompensated liver cirrhosis

Consent from the clinical research unit on the program:

I have read this program carefully. I agree with the inclusion of all the necessary information in the program to conduct the research, and I agree with the content described in the program. I understand that without the approval of the ethics committee, the trial must not be started, and the relevant regulations of the unit must be fully complied with.

Clinical research unit: No. 302 Hospital of the PLA

Signature: ____________________________ Date: ____________________

Clinical trial program confirmation signature page

Clinical research program of human mesenchymal stem cells in the treatment of decompensated liver cirrhosis

Consent from data manager and statistical analysts on the program:

I have read this program carefully. I agree with the inclusion of all the necessary information in the program to conduct the research, and I agree with the content described in the program. I understand that without the approval of the ethics committee, the trial must not be started, and the relevant regulations of the unit must be fully complied with.

Unit of data management and statistical analysis

Signature: ____________________________ Date: ____________________
